# Risks to Birds Traded for African Traditional Medicine: A Quantitative Assessment

**DOI:** 10.1371/journal.pone.0105397

**Published:** 2014-08-27

**Authors:** Vivienne L. Williams, Anthony B. Cunningham, Alan C. Kemp, Robin K. Bruyns

**Affiliations:** 1 School of Animal, Plant and Environmental Sciences, University of the Witwatersrand, Wits, South Africa; 2 School of Public Leadership, Stellenbosch University, Stellenbosch, South Africa; 3 Ditsong Museum of Natural History, Pretoria, South Africa; University of Kent, United Kingdom

## Abstract

Few regional or continent-wide assessments of bird use for traditional medicine have been attempted anywhere in the world. Africa has the highest known diversity of bird species used for this purpose. This study assesses the vulnerability of 354 bird species used for traditional medicine in 25 African countries, from 205 genera, 70 families, and 25 orders. The orders most represented were Passeriformes (107 species), Falconiformes (45 species), and Coraciiformes (24 species), and the families Accipitridae (37 species), Ardeidae (15 species), and Bucerotidae (12 species). The Barn owl (*Tyto alba*) was the most widely sold species (seven countries). The similarity of avifaunal orders traded is high (analogous to “morphospecies”, and using Sørensen's index), which suggests opportunities for a common understanding of cultural factors driving demand. The highest similarity was between bird orders sold in markets of Benin vs. Burkina Faso (90%), but even bird orders sold in two geographically separated countries (Benin vs. South Africa and Nigeria vs. South Africa) were 87% and 81% similar, respectively. Rabinowitz's “7 forms of rarity” model, used to group species according to commonness or rarity, indicated that 24% of traded bird species are very common, locally abundant in several habitats, and occur over a large geographical area, but 10% are rare, occur in low numbers in specific habitats, and over a small geographical area. The order with the highest proportion of rare species was the Musophagiformes. An analysis of species mass (as a proxy for size) indicated that large and/or conspicuous species tend to be targeted by harvesters for the traditional medicine trade. Furthermore, based on cluster analyses for species groups of similar risk, vultures, hornbills, and other large avifauna, such as bustards, are most threatened by selective harvesting and should be prioritised for conservation action.

## Introduction

The balance between culture, ritual, commerce and conservation is an emotive issue, particularly where charismatic animal species are used for traditional medicine (TM). For example, the expanding trade in animal derivatives, such as tiger bones, bear gallbladders and rhino horns in the Far East for Traditional Asian Medicine, is especially controversial and of international concern, since the species concerned are endangered yet in high demand [1]. The practice of healing using animals (‘zootherapy’) [2]–[4], however, has deep historical origins. Civilizations in Ancient Egypt, Mesopotamia and China have written records of therapies that require bat limbs, mongoose blood or glands from musk deer, respectively, and included in these archives from a bygone age are medicinal remedies using swallow's liver, bird excrement and chicken eggs [4].

**Figure 1:**
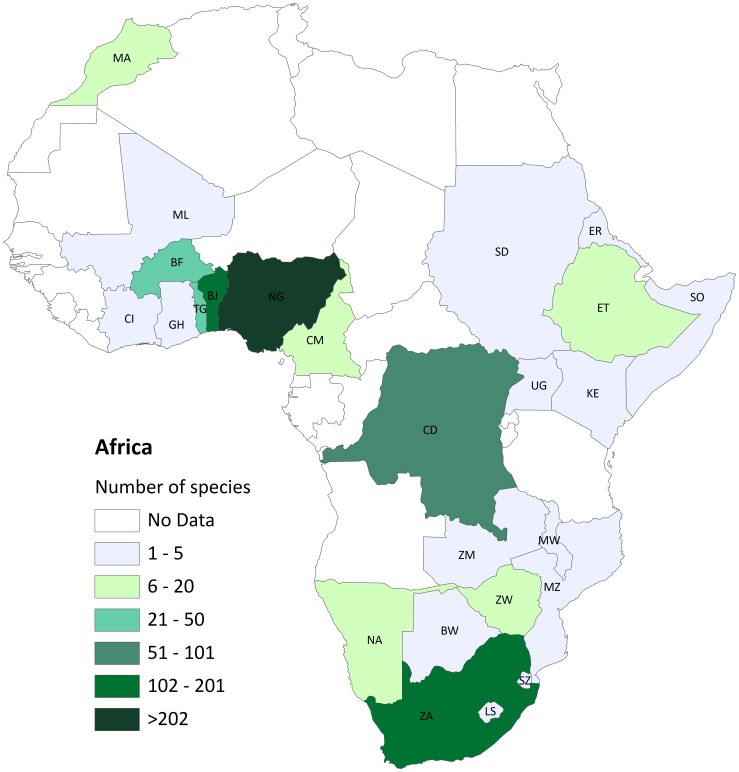
Bird species traded for traditional medicine. number of species across 25 African countries.

Indigenous knowledge (IK) related to the consumptive utilisation of avifauna span continents and cultures. At a global scale, at least 4,173 bird species (42% of 9,856 extant avian species) are used by people, mainly as pets (37%) or food (14%), with far fewer used in sport hunting, ornamentation or TM (<1%, see [5] Figure 1). In the early 1900s, various authors (e.g. [6]–[8]) documented customary uses and reverence for birds within indigenous cultures (e.g. *“Woe betide the native who…kills one of these birds; he will be struck down by…illness, which…will terminate in his death”* [8]), which stemmed from curiosity about ethno-ornithology and the indigenous use of avifauna. In more recent years, concerns have emerged about both the sustainability of utilisation and the need to document IK in the face of its apparent erosion due to modernization and adoption of western medicinal and cultural practices. Accordingly, researchers have turned their attention to inventorying species and documenting zootherapeutic practices [2], [9]–[11].

Growth in the number and size of markets for TM has been correlated with increased numbers and levels of species harvested [12]. As TM markets have grown, so has commercial availability of targeted taxa and concerns that preferred species are being acquired in an unsustainable manner. However, few studies have quantified the trade or evaluated the impact that unregulated commercial collection may be having on populations of threatened avian species. In Africa, most studies conducted to date are country-specific surveys of TM or fetish markets, or taxon-specific studies focussed on such birds as ground-hornbills, owls or vultures. Cocker & Mikkola [13], for example, suggested that harvesting may involve *“thousands, possibly tens of thousands, of owls annually”*. Bruyns *et al*. [14] reported on the sale of Southern Ground-hornbill (*Bucorvus leadbeateri*) at a market in Zimbabwe and concluded that since hunting for this species was mainly opportunistic, the medicinal trade was not likely to be a significant threat to its national population. National surveys have been carried out in Benin [15], Nigeria [16], [17], and South Africa [18]–[20]. One of the most comprehensive country surveys is of the quantities of bird species observed in each of 24 Nigerian markets, together with insights into how species are collected, priced and used, and anthropogenic factors that directly or indirectly influence their trade [17].

In response to the growing TM trade as a potential threat to wildlife in South Africa, Cunningham & Zondi [18] conducted one of the first ethno-zoological trade assessments in 1991. Vultures, Bateleur Eagles (*Terathopius ecaudatus*), and Southern Ground-hornbills were identified as conservation priorities. Concerns regarding the hunting of vultures for TM in South Africa, especially the near-endemic and threatened Cape Vulture (*Gyps coprotheres*), led Mander *et al*. [19] to quantitatively assess the vulture trade in South Africa, consulting traditional healers and making recommendations for conservation action. This, and Cocker & Mikkola's work on owls, identified priority conservation taxa in the TM trade [13], [19].

In contrast to these national studies, even fewer regional or continent-wide assessments have been conducted in Africa. Only Marshall's [21] report on medicinal wildlife resources in East and southern Africa incorporated a wide-ranging account of birds in TM and their associated use, as well as their trade and conservation statuses within the source countries. More recently, Williams *et al*. [22] (the authors of this paper) quantitatively assessed the richness and rarity levels of avifauna used and sold for TM within 25 African countries. In assessing rarity, the study was partly reliant on species-specific data published on the BirdLife International website [www.birdlife.org]. However, the 2012 update of the IUCN Red List for birds resulted in new and revised information being available for a large proportion of the species investigated, thereby rendering the book chapter out-dated by the time it was published. The current paper re-assesses the vulnerability of these avian taxa to harvesting on the basis of these more recent data, but also includes new analyses to account more comprehensively for the richness and prevalence of avian taxa sold in African TM markets, substantially updating Williams *et al*. [22]. Our objectives were to: i) update the list of avian species recorded in TM markets using published accounts and personal observations; ii) re-examine patterns of rarity and commonness among avifauna used; iii) relate mean body size (mass) to inherent rarity and the prevalence of species in the markets, and iv) detect taxa that may be vulnerable to selective harvesting, in conjunction with current and potential threats to their existence, and which may arise from any escalation in their use and commercial harvesting.

## Methods

### Data Sources

**Figure 2:**
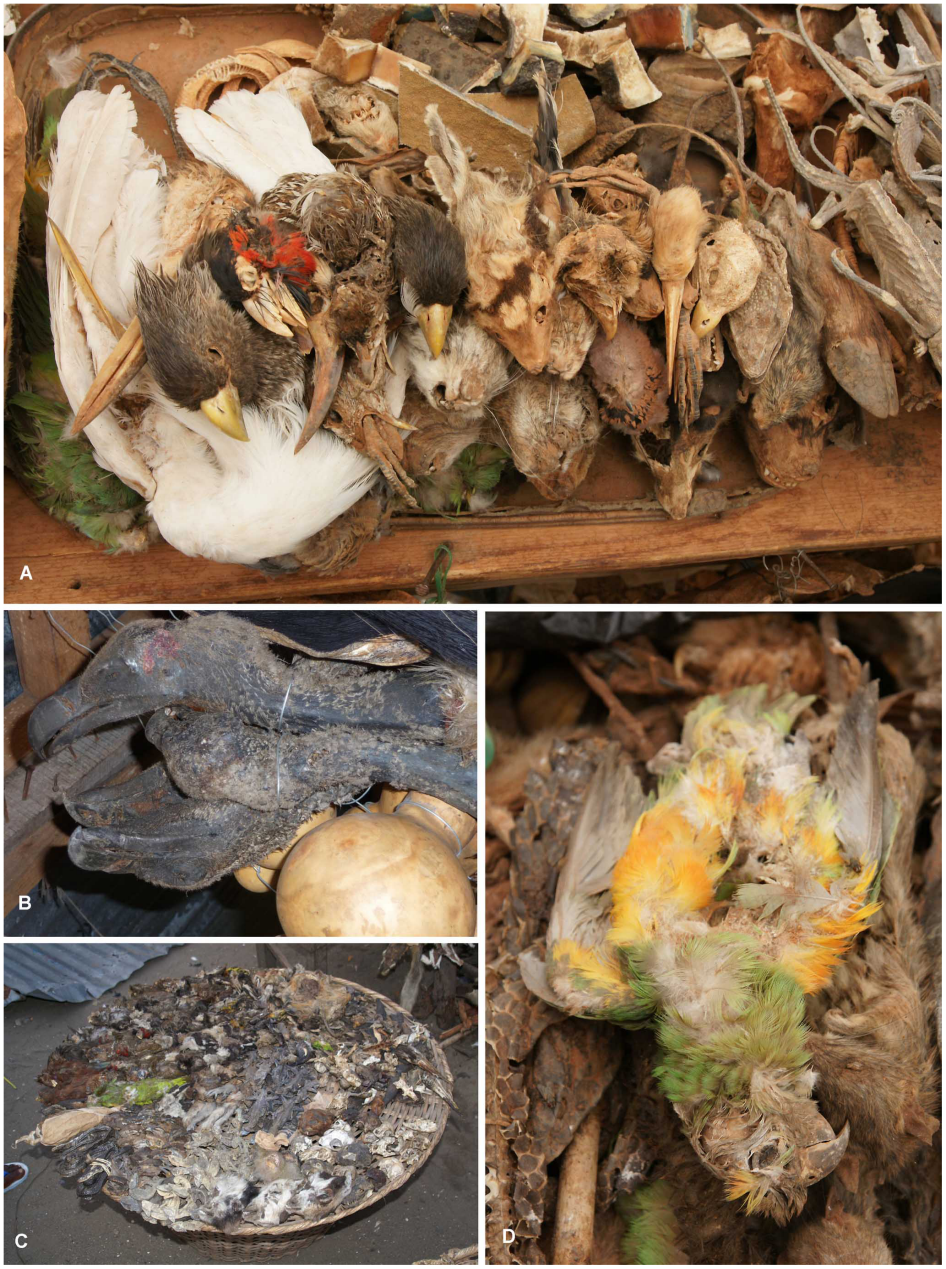
African bird species in the traditional medicine trade. **A**. Heads of a variety of species, including a Western Grey Plantain-eater (*Crinifer piscator*), Double-toothed Barbet (*Lybius bidentatus*), Red-billed Hornbill (*Tockus erythrorhynchus*) and Double-spurred Francolin (*Francolinus albogularis*) (Ouagadougou market, Burkina Faso). **B**. Vultures and raptors, a high conservation priority group, sold here in Xipamanine market, Maputo, Mozambique. **C**. A basket of more than 15 species, including Broad-billed Roller (*Eurystomis glaucurus*), Fine-spotted Woodpecker (*Campethera punctuligera*), African Wood-Owl (*Strix woodfordii*), Rose-ringed Parakeet (*Psittacula krameri*), White Helmet-Shrike (*Prionops plumatus*), and Standard-winged Nightjar (*Macrodipteryx longipennis*) (Dantokpa market, Benin). **D**. Senegal Parrot (*Poicephalus senegalus*) (Ouagadougou market, Burkina Faso). [Photos: A.B. Cunningham]

The inventory of birds recorded in TM markets and shops in Africa (Figure 1) was compiled from published accounts [13], [15]–[19], [21]–[46], and supplemented by our own personal research, observations and photographs taken in various TM markets across Africa over 23 years (1989–2012). The most comprehensive published information available was for birds sold in TM markets in Benin (BJ) [15], Nigeria (NG) [17], and South Africa (ZA) [22]. In addition, market data for BJ (Dantokpa market), Burkina Faso (BF; Ouagadougou), Côte D'Ivoire (CI; Abidjan and Bouake), Togo (TG; Lome market), ZA (Johannesburg) and Zimbabwe (ZW; Bulawayo) were supplemented with identifications made from photographs taken during fieldwork (Figure 2). However, we do not consider our inventory to be complete for Africa since the information was patchy and there was a paucity and/or absence of information for certain regions, especially East Africa (Figure 1). Hence, some of the findings must be viewed in light of these information gaps. The complete inventory for 399 taxa is published in Table S1.

### Ornithological Classification and Enumeration

The classification and nomenclature of BirdLife International was followed because their taxonomic list is kept current and forms the basis for the IUCN Red List assessments (along with Tobias *et al*. [47] for the forthcoming checklist of the birds of the world [48]). Furthermore, it was important that the quality of quantitative information obtained for the majority of species was consistent and derived primarily from the same source. While the BirdLife ornithological classification differs slightly from other taxonomic lists available online, it was necessary to adhere to one system. This meant, however, that five taxa considered separate species in some ethno-avian literature were ‘lumped’ with other species for this paper, namely: (i) Burchell's Coucal (*Centropus burchellii*) with White-browed Coucal (*Centropus superciliosus*) (ZA, BJ); (ii) Dark-capped Bulbul (*Pycnonotus tricolor*) with Common Bulbul (*Pycnonotus barbatus*) (BJ, NG, ZA); (iii) Sahel Paradise-Whydah (*Vidua orientalis*) with Eastern Paradise-Whydah (*Vidua paradisaea*) (NG); (iv) Cape White-eye (*Zosterops virens*) with Pale White-eye (*Zosterops pallidus*) (ZA); and (v) African Hoopoe (*Upupa africana*) with Eurasian Hoopoe (*Upupa epops*) (BF, NG, SD, ZA and Morocco, MA), although the BirdLife Taxonomic Working Group is reviewing the latter treatment. Taxonomic data and IUCN Red-Listing are correct to June 2014.

We were conservative in our enumeration of the total number of avian taxa. If a bird could not be recognized beyond genus, it was not included in any further analyses. Also excluded, except where specified, were:

- Two exotic species recorded in the markets, namely Indian Peafowl (*Pavo cristatus*) and Common Myna (*Acridotheres tristis*) since, while these species might be used now, they would not have formed part of traditional inventories;
- 15 species of Palearctic (PAL) non-breeding migrants to Africa (Common Cuckoo, Spotted Flycatcher, Montagu's Harrier, Pallid Harrier, Northern House-martin, Red-necked nightjar, Northern Pintail, Kentish Plover, European Roller, Great Snipe, Jack Snipe, White Stork, Barn Swallow, Yellow Wagtail, Eurasian Wryneck) (respectively, *Cuculus canorus, Muscicapa striata, Circus pygargus, Circus macrourus, Delichon urbicum, Caprimulgus ruficollis, Anas acuta, Charadrius alexandrinus, Coracias garrulus, Lymnocryptes minimus, Ciconia ciconia, Hirundo rustica, Motacilla flava, Jynx torquilla*). The PALs were omitted because much of the data used to assess threats to African species would not apply (even for the few species with a small breeding range extending into the Palearctic extremities of North Africa or into southern Africa), complicated further by estimating the extent of their actual wintering range in Africa versus areas visited in transit to and from the Palearctic summer breeding range. Note that there are other intra-African migrant species with separate breeding and non-breeding ranges but, since both ranges fall within the Afrotropics, they are not considered separately from other Afrotropical species. However, given the substantial declines that PALs have undergone in the last 30 years [49], [50], we have discussed the findings for PALs separately;
- 49 species recorded as being used for TM but not recorded in the TM markets, since our analyses are based on species selectively harvested or acquired for the commercial trade.

The richness and percentage-similarity of species and orders sold in markets in various African countries were compared using the Sørenson Index (for incidence-based data), calculated using EstimateS (version 7.5.1) [51].

### Patterns of rarity and commonness

One way of examining avian vulnerability to consumptive use is to classify the species based on the probability of them becoming rare if exploitation and persistent, selective, commercial hunting become deterministic factors that threaten population dynamics. Rabinowitz developed the ‘seven forms of rarity’ model that was originally applied to assess the vulnerability of plants [52], [53]. The model was based on three variables that indicated the level of rarity, namely: range size, habitat specificity and local abundance (Table 1). When species are dichotomized for each of the variables, the result is an eight-cell model (Table 1) that Yu & Dobson [54] adapted and used to create four ranks of rarity for assessing the rarity and commonness of mammals. For example, Category H species are rare in all three factors and assigned a rank of 1, whereas Category A species are common and assigned a rank of 4.

**Table 1:** Rabinowitz's 7 forms of rarity based on three traits.

Geographic Range		*Large*		*Small*	
Local population size		*Large, dominant somewhere*	*Small, non-dominant*	*Large, dominant somewhere*	*Small, non-dominant*
**Habitat specificity**	*Wide*	(A) Locally abundant in several habitats over a large geographic area (**4**)	(C) Constantly sparse in several habitats over a large geographic area (**3**)	(E) Locally abundant in several habitats over a small geographic area (**3**)	(G) Constantly sparse in several habitats over a small geographic area (**2**)
	*Narrow*	(B) Locally abundant in a specific habitat over a large geographic area (**3**)	(D) Constantly sparse in a specific habitat over a large geographic area (**2**)	(F) Locally abundant in a specific habitat over a small geographic area (**2**)	(H) Constantly sparse in a specific habitat over a small geographic area (**1**)

Letters in brackets indicate the rarity class, whereas numbers in bold in brackets indicate the ranks assigned to each rarity class. [Adapted from 52,54,99]

Yu & Dobson's [54] classes of rarity were applied to the data by placing each species in a category ranging from A to H. Thereafter, the categories were assigned ranks from 1 to 4 (most to least rare respectively; Table 1). The purpose was to examine patterns of rarity and commonness, comparing these classifications quantitatively with other variables to detect taxa that may be vulnerable to the TM trade. The data used to do the rarity assessments were mostly obtained from the BirdLife International website (http://www.birdlife.org/datazone/species), including: (i) estimates of population size, (ii) population trends (i.e. increasing, decreasing, stable), (iii) Extent of Occurrence (EOO, km^2^), (iv) number of Level 1 habitats, and (v) number of African countries in which the species occur. Some habitat and population abundance (‘status’) assessments were validated against information obtained from Sinclair & Ryan [55]. All these data were correct as of December 2012.

Assigning species to the rarity categories requires dichotomizing the distribution (large or small), habitat (broad or narrow) and population abundance (large/high/dominant or small/rare/non-dominant). For the distribution range, the median EOO for all the inventoried species was determined to be 6,790,000 km^2^ (range: 59,500 km^2^ to 63,300,000 km^2^; *n* = 346) and therefore EOOs greater and smaller than the median were considered to be large and small ranges respectively. Dichotomizing habitat and abundance were more subjective, since habitat types are essentially various graded combinations of geological, topographical, climatic, aquatic and vegetation features, ranging from desert to rainforest, and abundance ranges from rare to abundant. Sinclair & Ryan [55] were used to assist in borderline judgments.

One-way ANOVAs were computed to test the differences in the mean EOOs for species sold in markets that were assigned to the rarity groups ranked from 1 (most rare) to 4 (most common). The significance between group means was determined using the post-hoc Tukey HSD multiple comparison test for unequal *N* using Statistica 6 (Statsoft Inc).

### IUCN Red List Status

The IUCN status and threats to avian diversity were further examined based on the statuses available on the IUCN Red List of Threatened Species (www.iucnredlist.org, downloaded December 2012 and June 2014) and BirdLife International websites. The Red List status of a species will indicate whether they are likely to be increasingly threatened if continuous high-impact harvesting occurs.

### Body Size and Mass

Given evidence that extinction risks to birds incurred through human persecution was correlated with large body size [56], we calculated and compared the mean mass of traded birds in the different rarity classes and IUCN threat categories, as well as birds specifically sold at markets in ZA, BJ and NG. Mean body mass was obtained for 344 species from the BirdLife International website and for southern Africa from Chittendon & Upfold [57]. Mass was used as a proxy for size because mass is used in scaling analyses that predict that larger birds will occur at lower densities, have lower recruitment rates, live longer and therefore be more vulnerable to harvesting [58]. The average mass of the Ostrich (*Struthio camelus*) (114 kg) was excluded from all analyses since the species is a statistical outlier by more than three standard deviations when regressed against body length (*r* = 0.51 for *n* = 203 including Ostrich; *r* = 0.84, *p*<0.001, *n* = 202 excluding Ostrich).

### Cluster Analysis and Conservation Priorities

Cluster analysis is an effective way of identifying groups of species with profiles of similar risk and/or conservation priority in relation to criteria chosen to characterise these risks [59]–[64]. One purpose of a cluster analysis is to partition objects (such as species) into groups suggested by the data rather than defined *a priori*, so that objects in a given cluster tend to be relatively similar to each other and objects in different clusters are dissimilar [65]. In ethno-ecological studies, determinations of conservation priorities are regularly made using linear numerical rating systems, whereby ranked values are assigned to species for the different variables chosen. This method imposes artificial linearity onto a naturally non-linear system, tends to rank many species together and makes objective separation of species into priority hierarchies difficult [59]. Since the selection of hierarchical boundaries is often subjective or arbitrary, a recommended approach is to generate importance values based on ranks assigned within variables, sorting species in order of the variable values (or, total score) to place similar species nearby in ‘multivariate space’ [62], and using this order when conducting a cluster analysis [64].

*K-means clustering* is a simple non-hierarchical classification method appropriate as a data-reduction technique where there are large numbers of species, no real dependent variable and it is desirable to determine whether groups of similar species exist [64], [66]. Since this study is based on an inventory that we do not consider to be complete, and it was not possible to collect data for all the potential variables for all species, we selected only three variables for which we could obtain data for as many species as possible (280 of the 306 species recorded in the markets) so as to make a generalised assessment of priority and vulnerability.

The variables selected for the cluster analyses were: i) the four ranks assigned to the Rabinowitz rarity classes (since the system classifies species based on habitat specificity in addition to range and population size) (after Table 1); ii) mean body mass (since size was found to be related to vulnerability); and iii) the number of countries that recorded a species in at least one market (this, however, depended on the number of studies from which we could extract information). Since the variables were not all measured on the same numerical scale (e.g. body mass is continuous, and number of markets is discontinuous), the variable values were standardized and similarly scaled. The data were converted to scores per variable of between 0 (the lowest score and least vulnerable) and 1 (the highest score and most vulnerable). To do this, the values for each species in a corresponding variable for body mass and number of markets was divided by the highest value in that column but, for the rarity rank, the values first had to be reversed before being standardised since the original rank of 4 implied commonness and not rarity. The total scores for the three variables were summed, and the species arranged in descending order from highest to lowest scores (maximum score  =  three). Statistica 6 was used to perform the K-means cluster analyses. The process was repeated by progressively specifying the formation of two to four clusters and then evaluating the species within each cluster, ending up with only two groups of ‘higher’ (*S* = 115) and ‘lower’ (*S* = 165) conservation priorities and vulnerability (Table S2).

## Results

### Avifaunal Richness

We recorded 399 avian taxa as being used and sold for TM in 25 African countries (Tables 2 & S1), of which 354 were identified to species (Table 2). The species were from 207 genera, 70 families and 25 orders (Tables 3). When 49 species not recorded in the markets were excluded from the list, then the total ornithological richness of birds sold in the markets was 306 species from 189 genera, 69 families and 25 orders (including PALS and exotics) from 7 African countries (BF, BJ, CI, NG, TG, ZA and ZW) (Table 3).

**Table 2:** The number of identified and unidentified avian taxa used for traditional medicine between 25 African countries.

	Sold in markets (7 countries)	Not recorded in markets (18 countries)	Total taxa (25 countries)
Taxa identified to species	306^a^	49	354
Taxa identified as far as genus	16	2	18
Taxa identified as far as family	25	2	27
Total taxa	347	53	399

a 288 species after the exclusion of PALs and exotics.

**Table 3:** Summary of the number of avian taxa per order used and sold for traditional medicine. The totals exclude the 45 unidentified taxa.

	All species used			Species sold in the markets	
Order (22)	No. families per order (*S* = 70) a	No. genera per order (*S* = 207)	No. species per order (*S* = 354) b	No. genera per order (*S* = 189)	No. species per order (*S* = 306) b
Anseriformes (Waterfowl)	1	5	7	5	7
Apodiformes (Swifts & relatives)	1	1	2	1	2
Bucerotiformes (Hornbills)	2	5	14	5	14
Caprimulgiformes (Nightjars & relatives)	1	2	5	2	3
Charadriiformes (Gulls & relatives)	7	12	19	12	19
Ciconiiformes (Storks)	2	15	20	15	20
Coliiformes (Mousebirds)	1	1	1	1	1
Columbiformes (Doves & pigeons)	1	5	10	5	9
Coraciiformes (Kingfishers & relatives)	5	11	24	11	23
Cuculiformes (Cuckoos & relatives)	1	5	13	5	11
Falconiformes (Diurnal birds of prey)	2	29	45	27	43
Galliformes (Gamebirds)^d^	2	7	13	6	10
Gruiiformes (Cranes & relatives)	4	12	17	12	16
Musophagiformes (Turacos)	1	4	8	4	7
Passeriformes (Perching birds) d	23	61	107	50	79
Pelecaniformes (Pelicans & relatives)	4	6	8	6	8
Piciformes (Woodpeckers & relatives) a	3	9	15	6	10
Podicipediformes (Grebes)	1	1	1	1	1
Procellariiformes (Albatrosses & relatives)	1	1	1	1	1
Psittaciformes (Parrots)	1	4	6	4	5
Pteroclidiformes (Soundgrouses)	1	1	1	1	1
Sphenisciformes (Penguins)	1	1	1	1	1
Strigiformes (Owls)	2	7	14	6	13
Struthioniformes (Ratites)	1	1	1	1	1
Trogoniformes (Trogons & relatives)	1	1	1	1	1

a the Family Indictoridae (Honeyguides) are absent from the traded species list, hence *S* = 69 traded families;

b includes migrant Palearctic (PAL) bird species;

d includes 1 exotic species.

Perching songbirds (Passeriformes), which comprise 56% of Africa's bird species (inferred from BirdLife International website, 2013), had the highest number of recorded taxa in use (23 families, 61 genera, 107 species) and in trade (50 genera, 79 species) (Table 3). Within the traded Passeriformes, the Sturnidae (starlings) was the most prevalent family (9 species). Of all the families in TM trade, the Accipitridae (Order Falconiformes) had the most recorded genera (26 genera; 37 species; including kites, hawks, eagles, vultures), followed by the Ardeidae (Order Ciconiiformes) (11 genera; 15 species; including herons and egrets) (Table S1). The Bucerotidae (hornbills), Cuculidae (cuckoos) and Strigidae (owls) were the next most specious families in trade (12, 11 and 11 species per family, respectively).

The highest number of species were recorded in NG markets (200 species, plus one exotic, five PAL and six unidentified species), followed by BJ (134 species, plus nine PAL and nine unidentified), ZA (84 species, plus two exotic and 24 unidentified), BF (29 species, plus one PAL and 11 unidentified), CI (12 species, plus three unidentified), TG (11 species, plus 13 unidentified) and ZW (six species, plus six unidentified) (Figure 1). Fourteen PAL species were only recorded in NG, BJ and BF markets. The Red-necked Nightjar was only recorded as being used (not traded) in MA. Five intra-African migrants were also identified (Wahlberg's Eagle, Bronze-winged Courser, Levaillant's Cuckoo, Violet-backed Starling, African Golden Oriole; respectively *Aquila wahlbergi*, *Rhinoptilus chalcopterus*, *Clamator levaillantii*, *Cinnyricinclus leucogaster*, *Oriolus auratus*), but only in NG and BJ markets, despite their total range in Africa extending to southern Africa.

### Frequency and Similarity of Species Traded in Markets

Ostriches were the most commonly used of all the birds (12 countries), but the species has only been positively identified in the markets of four countries to date (MA, NG, ZA, ZW) (Table 4). The most frequently recorded species in markets were the Barn Owl (seven countries), Pied Crow (*Corvus albus*; six countries), and Hooded Vulture, Helmeted Guinea Fowl and African Pied Hornbill (*Necrosyrtes monachus*, *Numida meleagris*, *Tockus fasciatus*, respectively; five countries each) (Table 4). Of the top 19 species, only three are currently threatened and the populations of six species are experiencing declines in numbers (Table 4). Fifteen of the top 19 species are, however, widespread with EOOs >7 million km^2^. The EOO of the Barn Owl is estimated to be 63 million km^2^ [67], making it one of the species most likely to be selected for TM use given its cultural significance and spatial commonness. The African Pied Hornbill, Western Grey Plantain-eater (*Crinifer piscator*) and Grey Parrot (*Psittacus erithacus*), despite having ranges restricted to <4.5 million km^2^ and occurring in specific habitats, are locally abundant species frequently available in West African markets.

**Table 4:** The most frequently recorded species in African countries and markets, excluding unidentified morphospecies (such as ‘eagle’ or ‘*Tockus* sp.’).

Common name	Species	No. countries use reported in (*n* = 25)	No. countries reporting market observations (*n* = 7)	2014 IUCN Red List Status a	Population trend b
Owl, Barn	*Tyto alba*	7	7	LC	S
Crow, Pied	*Corvus albus*	8	6	LC	I
Vulture, Hooded	*Necrosyrtes monachus*	6	5	EN	D
Guineafowl, Helmeted	*Numida meleagris*	6	5	LC	S
Hornbill, African Pied	*Tockus fasciatus*	5	5	LC	?
Ostrich	*Struthio camelus*	12	4	LC	D
Roller, Abyssinian	*Coracias abyssinicus*	5	4	LC	I
Wood-owl, African	*Strix woodfordii*	5	4	LC	S
Egret, Cattle	*Bubulcus ibis*	4	4	LC	I
Plantain-eater, Western Grey	*Crinifer piscator*	4	4	LC	S
Fish-eagle, African	*Haliaeetus vocifer*	4	4	LC	S
Night-heron, Black-crowned	*Nycticorax nycticorax*	4	4	LC	D
Hornbill, Red-billed	*Tockus erythrorhynchus*	4	4	LC	S
Parrot, Grey	*Psittacus erithacus*	6	3	VU	D
Eagle-owl, Spotted	*Bubo africanus*	5	3	LC	S
Ground-hornbill, Abyssinian	*Bucorvus abyssinicus*	5	3	LC	S
Hamerkop	*Scopus umbretta*	5	3	LC	S
Hoopoe, Eurasian	*Upupa epops*	5	3	LC	D
Ground-hornbill, Southern	*Bucorvus leadbeateri*	5	2	VU	D

a EN = Endangered; VU = Vulnerable; NT = Near Threatened; LC = Least Concern.

b S = Stable, I = Increasing, D = Decreasing;? unknown (from BirdLife website).

The countries most similar in terms of the species sold in the markets are BJ vs. NG (53% similar in terms of Sørenson's index; 93 species in common), followed by NG vs. ZA (27% similar, 39 species in common) and BJ vs. ZA (24% similar, 28 species in common) (Table 5). BJ, NG and ZA also have 23 species in common between them. Despite the limited survey work conducted in BF, the species sold in the markets there are 24% similar to those sold in BJ.

**Table 5:** Comparisons of the percentage similarity of species and orders of birds sold at different markets, showing the low similarity at the species level and high similarity at the order level.

		Sørenson's % similarity	
Country A	Country B	Species sold	Orders (morphospecies) sold
Nigeria	Benin	53%	87%
South Africa	Nigeria	27%	81%
Benin	Burkina Faso	24%	90%
South Africa	Benin	24%	87%
Nigeria	Burkina Faso	21%	78%
South Africa	Burkina Faso	15%	77%

While there are geographical differences in the occurrence of species, and hence their occurrence in markets, the selection of birds for medicine is often at a less specific ‘morphospecies’ level (i.e. a typological 'species' that can only be identified as owl, vulture or kingfisher). In South Africa, for example, all vultures, regardless of species, are generically referred to in isiZulu as ‘*iNqe*’. When re-assessing the frequencies of birds in the markets of seven countries at the level of order (analogous to morphospecies), the most prevalent morphospecies identified were: 1) owls (Strigiformes), hornbills (Bucerotiformes), diurnal birds of prey (Falconiformes), perching birds (Passeriformes) and gamebirds (Galliformes) (seven countries each); 2) storks, herons and egrets (Ciconiiformes) and kingfishers (Coraciiformes) (six countries each); and 3) turacos (Musophagiformes), woodpeckers and relatives (Piciformes), doves and pigeons (Columbiformes), pelicans and relatives (Pelicaniformes) cranes and bustards (Gruiformes) (five countries each). Using Sørenson's similarity index, we also assessed the similarity of avifaunal orders of birds sold in the markets (Table 5). The highest similarity was between bird orders sold in markets of BJ vs. BF (90%), followed by NG vs. BJ (87%). Even bird orders traded in two geographically separated countries (BJ vs. ZA, and NG vs. ZA respectively) were 87% and 81% similar.

### Rabinowitz Classification and Patterns of Rarity and Commonness

**Figure 3:**
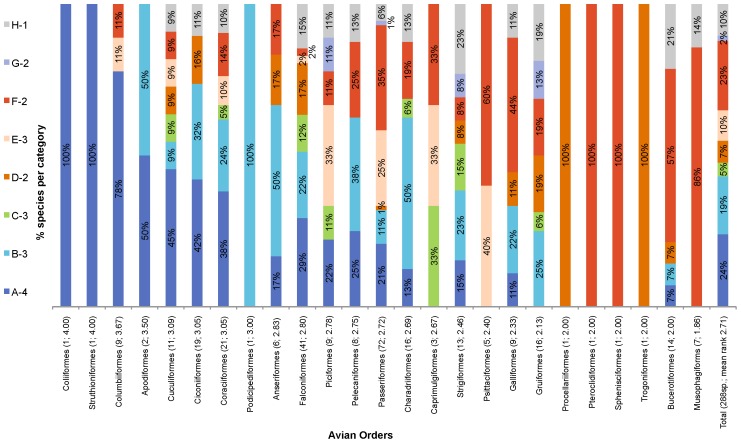
The proportion of traded bird species per order in the eight Rabinowitz classes (excluding PALs and exotics). The number of species per order and the mean rarity rank (derived from rank scores in Table 1) are given in parentheses. The orders are listed from most common to most rare (left to right respectively).

The number and proportion of species varied within the categories of rarity (Table 6) and within the rarity categories per order (Figure 3). About 23% (69 species) of birds sold in the markets are very common and locally abundant in several habitats over a large geographic area (Category A), whereas 10% (30 species) were in the most rare category and are considered to be consistently sparse in a specific habitat over a small geographic area (Category H) (Table 6).

**Table 6:** IUCN Red List status, rarity categories and population trends for traded species, where species in categories A and H are least and most rare respectively (excluding PALs and exotics).

	IUCN Red List Status a				Total N	Population trends			
Rarity	EN	VU	NT	LC		Stable	Decreasing	Increasing	Unknown
A	1			68	69	37	15	15	2
B			1	54	55	24	16	8	7
C	3			10	13	7	5	0	1
D		4	2	15	21	8	11	0	2
E				29	29	19	6	3	1
F	1	3		62	66	36	18	2	10
G				5	5	3	1	0	1
H		5	2	23	30	13	14	0	3
Total	5	12	5	266	288	147	87	28	19
%	1.7%	4.2%	1.7%	92.4%	-	51.0%	29.9%	9.7%	9.4%

a EN = Endangered; VU = Vulnerable; NT = Near Threatened; LC = Least Concern.

Species within orders were not homogeneously distributed among the categories of commonness and rarity (Figure 3). Compared to other orders, Strigiformes (owls) had the highest proportion of rare species in Category H (23%), followed by Bucerotiformes (hornbills; 21%), and Gruiiformes (cranes; 19%) (Figure 3). Conversely, Columbiformes (doves and pigeons) had the highest proportion of common species (Category A; 78%). While 23% of the traded owls tend to be constantly sparse in specific habitats over a small geographic area (Category H), an equal proportion is locally abundant in specific habitats over a large geographic area (Category B; Figure 3). Hence, when the mean rarity rank per order was calculated (based on Table 1, with ranks closer to 1 indicating greater relative rarity), the orders that were relatively rarer and had a higher proportion of rare species (excluding those with less than five species), were Musophagiformes (mean rank = 1.86; S = 7 species), Bucerotiformes (mean rank = 2.00; *S* = 14 species), Gruiformes (mean rank = 2.13; *S* = 16), and Galliformes (gamebirds; mean rank = 2.33; *S* = 9) (Figure 3). The orders with the highest proportion of common species (i.e. category A) are Columbiformes (mean = 3.67; *S* = 9), Cuculiformes (cuckoos and relatives; mean = 3.09; *S* = 11), Coraciiformes (kingfishers and relatives; mean = 3.05; *S* = 21) and Ciconiiformes (storks; mean = 3.05; *S* = 19) (Figure 3). The overall mean rank for all traded species was 2.71 (*S* = 288 species), indicating that there is an intermediate degree of inherent commonness among the species sold for TM across the African continent.

**Figure 4:**
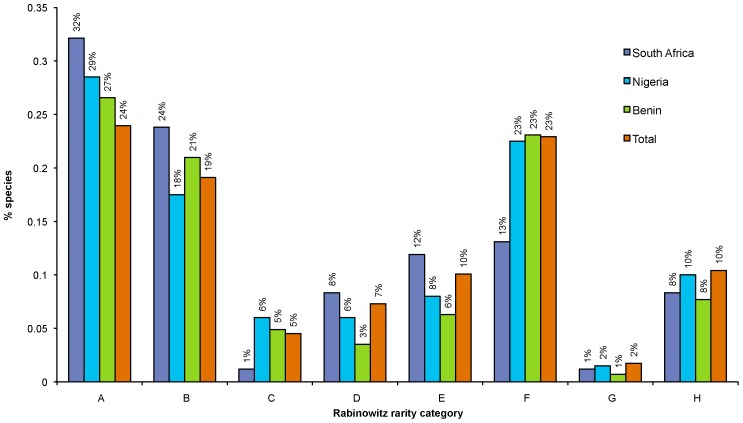
The observed proportion of traded species in each Rabinowitz category of commonness or rarity for two West African countries (Benin and Nigeria), for South Africa and the ‘Total’ for seven countries combined.

When comparing the observed number of traded species in the eight rarity categories for ZA, NG and BJ (Figure 4), broadly similar proportions of species were allocated to each rarity category. The major differences were, however, that ZA has a smaller proportion of species that are constantly sparse in several habitats over a large geographic area (category C) and a smaller proportion of species that are locally abundant in a specific habitat over a small geographic area (category F) – this is partly because its southern position supports fewer ‘typical’ African habitats, and its range-restricted species are of less favoured types. NG and BJ, however, utilise a larger proportion of species with narrower distribution ranges (categories E to F) – which is partly the result of harvesting species that inhabit the more range-restricted forest and woodland habitats that occur within the harvesting catchment available to the hunters and consumers in those countries.

When examining the individual factors that contribute towards the rarity of avian species sold for TM (Table 7), we found that 45% of species have ‘small’ geographic ranges (i.e. <6,790,000 km^2^), 24% are non-dominant or constantly sparse within their range, and 60% are quite habitat specific. Chi-squared pair-wise comparisons of the characteristics of rarity indicated range and habitat specificity to be significant factors in the allocation of species to categories (χ^2^ = 19.7, d.f. = 3, *P*<0.01). However, habitat specificity in combination with population size were nearly significant factors (χ^2^ = 7.6, d.f. = 3, *P* = 0.055), but range and population size combined were not significantly related to categorizations of rarity (χ^2^ = 1.1, d.f. = 3, *P* = 0.77).

**Table 7:** Number and percentage of traded species dichotomized according to their distribution range, population size and habitat specificity factors (excludes PALs and exotics).

Factor			
Distribution range a		No. of species (*S* = 288)	Percentage
Large	Geographic area EOO>6,790,000 km^2^	158	55%
Small	Geographic area EOO<6,790,000 km^2^	130	45%
**Population**			
High	Dominant somewhere/locally dominant	219	76%
Low	Non-dominant/constantly sparse	69	24%
**Habitat**			
Broad	Several habitats	116	40%
Narrow	Specific habitats	172	60%

a 6,790,000km^2^ is the median EOO for all the species investigated.

An EOO of ≤20,000 km^2^ is the quantitative threshold for classifying a species as threatened, specifically Vulnerable, according to the B1 Red List criterion of the IUCN. However, since birds are rarely range-restricted in the same sense and to the same extent that similarly threatened terrestrial animals and sessile plants are, the smallest range for a traded species in this study was five times larger than the B1 threshold (Knysna Turaco – *Tauraco corythaix*; 125,000 km^2^). Hence, the Rabinowitz classification offers an alternative method for evaluating vulnerability where the B1 criterion cannot be applied. The mean EOO of species within each rarity class decreased with increasing species rarity (Table S3). The mean EOO for all traded species was 9,897,230 km^2^ (*S* = 283), well above the 6,79 million km^2^ median for all species considered in this paper (Table S3).

### IUCN Red List Status and Population Trends

The IUCN Red List statuses of 17 traded species are threatened (five Endangered; 12 Vulnerable), five are Near Threatened (excluding PALs), and the majority of species (92%) are categorized as Least Concern (Table 6). For the most part, the populations of traded species are stable (51%) and/or increasing (10%; Table 6). However, populations are declining for 30% of species. Major threats to birds vary with species [56], [68] so, in devising conservation strategies, it is important not to focus unnecessarily on the TM trade.

### Body Mass and Rarity

**Figure 5:**
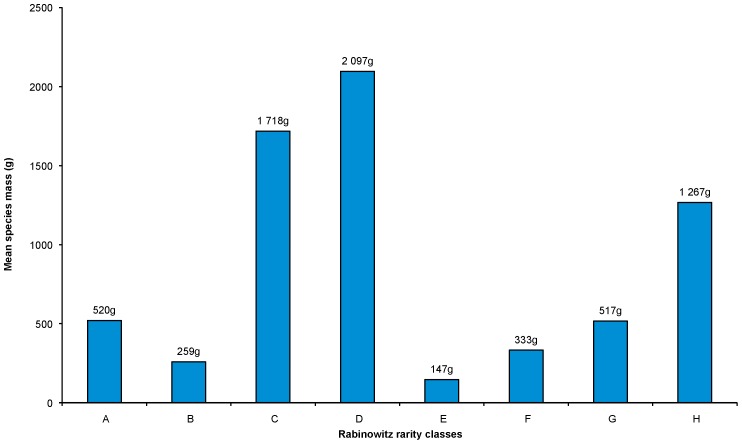
Mean mass (g) of bird species sold for traditional medicine within each of the eight Rabinowitz rarity classes.

From published accounts of birds sold in TM markets throughout Africa, there is evidence to suggest that larger birds have a greater tendency to be selectively harvested and are accordingly more prevalent in the markets [17], [18], [21]. Consequently, larger birds are at greater risk of population decline and localised extirpations. When analysing the mean mass of species in the eight rarity classes (excluding the Ostrich in class A), we found that larger birds tended to be non-dominant species (classes C,D,G,H; Figure 5), especially those occurring in specific habitats (classes D and H), whereas smaller birds tended to be locally abundant (classes A,B,E,F; Figure 5). When the factors are dichotomised (Table 8), the largest birds are usually those that occur over a large range (mean mass = 773 g), but have smaller population sizes and/or lower densities (mean mass = 1584 g) and occur in specific habitats (mean mass = 702 g) – the latter two factors increasing the risks of population declines with increased and selective utilisation of larger birds. When comparing the mean body mass of taxa in the adapted four rarity ranks, there is an increase in mean mass with increased rarity: rank 4 (most common)  = 520 g±1,078 g (± S.D.); rank 3 = 684 g±1,510 g; rank 2 = 868 g±1,656 g; and rank 1 (most rare)  = 1,267 g±2,258 g. Further evidence for larger birds being more vulnerable than smaller birds was derived from the mean mass calculated according to the IUCN Red List categories. Whereas traded species classified as Least Concern weighed on average 464 g±1,035 g (*n* = 259), threatened (Endangered and Vulnerable) species sold in the markets were 8.3 times heavier (3,832 g±2,598 g, *n* = 17), and Near Threatened species were 8.8 times heavier (4,083 g±1,592 g, *n* = 5).

**Table 8:** Mean mass of birds sold for traditional medicine in markets dichotomized according to distribution range, population size and habitat specificity factors (excludes PALs and exotics).

Factor	Mean mass ± S.D.
**Distribution range**	
Large	773 g±1,517 g
Small	491 g±1,179 g
**Population**	
High	351 g±717 g
Low	1,584 g±2,290 g
**Habitat**	
Broad	562 g±1,265 g
Narrow	702 g±1,453 g
	
Mean mass all species	644 g±1,377 g

When analysing the mean mass of species sold in ZA, NG and BJ markets, a similar pattern to that in Figure 5 emerged for each country (Figure S1). Furthermore, birds sold in markets in ZA were, on average, larger than those sold in markets in BJ and NG (1,197 g, 657 g, and 618 g respectively). Reasons for the average size of birds being heavier/larger in South Africa compared to West Africa are likely to be related to the trend in the sizes of the species that occupy the forest and woodland habitats within the latter's harvesting and hunting catchment.

### Vulnerability to Selective Harvesting

Through cluster analysis, avian species of varying degrees of rarity and commonness were assigned to internally homogenous clusters (groups 1 and 2) that were indicative of their relative conservation priorities (Table S2). These groupings indicated vulnerability to selective harvesting by integrating the biological and ecological traits of species with their frequency in TM markets. Species in Group 1 are generally more vulnerable to selective harvesting since they are inherently larger, rarer birds with populations that tend to be decreasing (Table S2). These species are also less widespread (mean EOO = 4,406,281 km^2^±3,822,394 km^2^) and sold in fewer markets than those in Group 2. However, their rarity, rather than consumer preference, is also a factor in them being recorded in fewer markets. This group contained all the Musophagiformes (turacos), many Bucerotiformes (hornbills; 86%), and most of the Galliformes (gamebirds; 67%), Gruiformes (cranes and relatives; 67%) and Psittaciformes (parrots; 60%) (Table S2).

Species in Group 2 are generally less vulnerable to selective harvesting since they tend to be smaller and more common, with populations that are stable or increasing (Table S2). These species have also been recorded in relatively more markets, and have larger ranges (14,037,660 km^2^±10,816,510 km^2^). Group 2 contains most of the Columbiformes (doves; 89%), Ciconiiformes (storks; 78%), Coraciiformes (kingfishers and relatives; 76%), Cuculiformes (cuckoos and relatives; 73%), Pelicaniformes (pelicans and relatives; 71%), Falconiformes (diurnal birds of prey; 69%), and Passeriformes (perching birds; 59%) (Table S2). Strigiformes (owls) are spread between Groups 1 (54%) and 2 (46%), which is indicative of their varying degree of vulnerability depending on the species.

Vultures and hornbills, and other large avifauna such as eagles and bustards, are clearly the taxa most vulnerable to selective harvesting for the TM trade, and dominate the list of species with the highest total standardized variable scores (Table 9). Six of the nine vulture species recorded in TM markets across Africa are on our list of most vulnerable species (Table 9). These results confirm the observations of several studies that larger birds are more likely to be used for TM [17], [18], [21] and/or more threatened by human persecution [57]. Not only are they vulnerable to being utilised, but they also tend to have low population numbers and densities, and occupy more specific habitats – thus making them more likely to be naturally rare and constantly sparse in specific habitats within the harvesting catchments of hunters/harvesters/consumers.

**Table 9:** The top 19 conservation priority bird species in the African traditional medicine trade in 25 countries, ranked by numerical importance value and showing the assigned risk group.

Order	Species	Common name	Total importance score (max 3)	Risk group a
Falconiformes	*Gyps coprotheres*	Vulture, Cape	2.13	1
Gruiformes	*Ardeotis arabs*	Bustard, Arabian	1.95	1
Bucerotiformes	*Bucorvus abyssinicus*	Ground-hornbill, Abyssinian	1.86	1
Falconiformes	*Torgos tracheliotos*	Vulture, Lappet-faced	1.82	1
Gruiformes	*Balearica pavonina*	Crowned-crane, Black	1.81	1
Falconiformes	*Gyps rueppellii*	Vulture, Rueppell's	1.80	2
Pelecaniformes	*Pelecanus onocrotalus*	Pelican, Great White	1.79	2
Falconiformes	*Stephanoaetus coronatus*	Hawk-eagle, Crowned	1.67	1
Falconiformes	*Gyps africanus*	Vulture, White-backed	1.58	2
Falconiformes	*Trigonoceps occipitalis*	Vulture, White-headed	1.54	1
Falconiformes	*Gypaetus barbatus*	Lammergeier	1.52	1
Ciconiiformes	*Ardea goliath*	Heron, Goliath	1.52	1
Bucerotiformes	*Tockus fasciatus*	Hornbill, African Pied	1.49	1
Bucerotiformes	*Bucorvus leadbeateri*	Ground-hornbill, Southern	1.44	1
Charadriiformes	*Vanellus lugubris*	Lapwing, Senegal	1.44	1
Cuculiformes	*Ceuthmochares aereus*	Yellowbill	1.44	1
Gruiformes	*Neotis denhami*	Bustard, Denham's	1.42	1
Bucerotiformes	*Bycanistes cylindricus*	Hornbill, Brown-cheeked	1.41	1

a See Tables S2a,b for a list of all species per risk group and a description of the risks.

### Palearctic (PAL) Migrants to Africa

Palearctic migrant species are long-distance migrants that breed in Europe and North Africa but spend the boreal winter in sub-Saharan Africa [69], usually from October to April [55]. Fifteen PAL migrants were recorded in the study, 14 between the markets of BF, BJ, NG and ZA (Table S1). Except for the European Roller (*Coracias garrulous*) and Barn Swallow (*Hirundo rustica*), 12 species were only recorded once and mainly in Benin. The only species to occur in South African markets was the large White Stork (*Ciconia ciconia*).

The species were mostly classified in Rabinowitz categories A–C, and had a mean rarity rank of 3.0 – indicating that they are generally not rare. The mean EOO for PALs at 13,537,786 km^2^ is twice that of the median range for all the species recorded in this study. However, the population trends for all except the Jack Snipe (*Lymnocryptes minimus*) are recorded as decreasing, with three species being classified as Near Threatened. Hence, the species are undergoing notable declines. As with the non-PAL species recorded in this study, birds with larger ranges tend to be smaller (mean = 389 g, or 170 g if the White Stork is excluded from the calculation).

## Discussion

### Avifaunal Richness and Use in TM

Globally, there are about 10,064 bird species (extant and extinct) [70], [71], with 2,355–2,600 of these occurring in Africa, 145 of which are PAL migrants to Africa. Africa has 23% of the total global avifauna recognised as species by BirdLife International [70], [71], of which at least 354 species (17%) are used for TM across the continent. While there are only records for 306 species (13%) being sold in markets in seven countries, more species are utilised in more countries than have been reported in the literature. The authors have observed the remains of unidentified birds in the rural and urban markets of Mozambique, Malawi, South Africa, Swaziland and Zambia, and are aware of cross-border trade between several southern African countries, while a Nigerian study mentions observations of birds at markets in Guinea and the cross-border trade of vultures from Chad and Niger [17].

The paucity of information on bird utilisation in East Africa warrants further consideration. Is this a result of communities not using and trading avifauna in the same manner as communities in southern and West Africa, or is little ethno-ornithological research being conducted in the region? Our field observations suggest that East Africa is different with regards to bird species in trade. Their urban TM markets are tiny in proportion to city size compared to West and southern Africa, typically comprising small numbers of Maasai women vendors trading in a low diversity of plant species and with no bird species seen for sale. However, the lack of information does not necessarily infer that little trade in, or use of, birds occurs. An ornithologist currently working in Kenya indicated that Tanzania is the “*epicentre for this* [traditional healing] *and no one works there, relatively speaking. Tanzanian witchdoctors are famous even in Kenya…It's pretty hard finding publications on this in East Africa*” [Darcy Ogada, pers. comm. 14/02/2013]. Furthermore, Ogada & Kibuthu [72] report that the study of African owls, for example, is confounded by strong cultural prejudices associated with the use of owls for witchcraft, and that these collective prejudices have resulted in an almost complete lack of long-term studies, including in Kenya. What has emerged for the region is an escalating illicit trade in eggs of Mackinder's Eagle-owl (*Bubo capensis mackinderi*) from Kenya for ‘witchcraft’ and cancer, which are purportedly destined for Tanzania and the Middle East [73], [74]. Clearly, consumptive utilisation of avifauna for TM in East Africa is occurring – but cultural taboos, a shortage of people conducting research, and differences in utilisation, trade and market structures/demand are factors limiting access to and availability of information. These same factors may also apply to other African countries for which there is also a paucity of information on consumptive use.

Despite this East African anomaly, there is a greater prevalence and diversity of bird species used and sold at TM markets in Africa than have been recorded on any other continent. In South America, for example, research on zootherapeutic resources in Brazil has indicated that there are at least 54 bird species used in folk medicinal practices [9], [10], [75], [76] (which is one species more than the 53 species recorded in just one TM market in South Africa by [20]). The most specious orders used in Brazil that are also frequently found in African TM markets, are the Passeriformes, Galliformes, Columbiformes and Falconiformes (Table S4). Less specious in Brazil, but more common in African markets, are the Ciconiiformes and Piciformes. In Asia, there is a growing body of zootherapeutic studies being carried out among tribes across India that are reporting on the indigenous uses of animals for TM [11], [78]–[80]. Although the overall richness of bird species used in India is relatively low (at least 31 species recorded from 19 studies), avian taxa accounted for an average of 21% of the vertebrate species recorded per study. In common with Africa and South America, the most specious orders used in India are the Passeriformes and Galliformes (Table S4). However, unlike South America, there is also frequent utilisation of Strigiformes (owls), besides Bucerotiformes (hornbills) species that do not occur in the Americas, whereas much-used Tinamiformes (tinamous) only occur in the Americas.

The African continent is clearly a priority region for vulnerability assessments to take place since a larger proportion of the avifauna are utilised and sold for TM. At a national level, 26.6% (134) of Benin's 503 bird species, 23.6% (200) of the 848 bird species in Nigeria and 11.1% (84) of South Africa's 754 bird species are commercially traded for TM. By comparison, Brazil has 1,721 bird species and India 1,167 species [81], with use for TM respectively representing only 3.1% and 2.7% of the total number of bird species.

In contrast to India, no birds are recorded as commonly traded in Chinese TM [82], apart from one notable exception – the massive regional trade in swiftlet nests (*Collocalia fuciphagus* and *C. maximus*; Apodiformes) from Southeast Asia to China. These are marketed by such companies as Eu Yan Sang (www.euyansang.com) as a ‘health food’ and are sold in China as a luxury food (*yàn wō*), generally known as ‘bird's nest soup’. Trade in swiftlet nests are in the grey area between food and medicine [83], but this Asian trade is well studied and managed [84], [85] and many of the nests come from artificially constructed nesting sites [86].

### Risks to Frequently Traded Species in African Markets

The impact of the TM trade, and localised non-commercial medicinal consumption, on levels of avian vulnerability or resilience has rarely been quantitatively addressed. Cluster analysis and the Rabinowitz rarity model provided objective ways of assessing the vulnerability to selective harvesting for a large number of avifauna sold for TM by integrating biological and ecological traits with records of their trade.

Threatened species that are particularly targeted by TM hunters (e.g. vultures, ground-hornbills, nest-sealing hornbills and various eagles; Table 9) will experience more significant declines in population numbers if selective hunting persists. More recently, the illegal collection of Mackinder's Eagle-owl eggs for use in ‘witchcraft’ has become a major threat to the population in Kenya in [72], and locals were reportedly being paid in the range of USD300–500 per pair of eggs c.2011 [Darcy Ogada, pers. comm., 14/02/2013].

This study revealed that 24% of traded bird species are very common and locally abundant, in several habitats and over a large geographic area, and so are unlikely to be of conservation concern (‘A’, Table 6), while only 10% of species are in the category of most rare (H, Table 6). We also suggest that, compared to the multiple factors and mechanisms that threaten avian species (e.g. intrinsic biological features, habitat loss, deleterious farming practices, and other forms of utilisation) [5], [56], [87], [88], the TM trade plays a minor role in the decline of species. Even in the case of birds in families prone to extinction through human use that are also used for TM, such as penguins (Spheniscidae), and albatrosses and petrels (Procellariidae) [56], our impressions are that these birds probably died at sea before ending up on beaches. For example, the presence of such species as the Shy Albatross (*Thalassarche cauta*) in a South African market (Table S1) may be the indirect result of natural or unnatural events, such as bad weather or long-line casualties, rather than targeted harvesting events. Based on discussions with traditional healers, the use of these stranded seabirds is influenced by the ritual potency of what are, to the healers, unusual pelagic birds tossed out by the sea and an uncommon occurrence.

In their study of the ecological basis for extinction risks in birds, Owens & Bennett [56] made the important point that different bird lineages may follow different paths to extinction. One route is for large-bodied, slow-breeding bird species to become threatened when the fecundity-mortality balance is disrupted by, for example, human use or introduced predators. A second route is for ecologically specialized species to become threatened by habitat loss [56]. These risk factors also apply to birds used for African TM. But whereas Owens & Bennet [56] correlated extinction risks incurred by smaller birds with a high degree of habitat specialisation, we show in Figure 5 and Table 8 that larger birds used for TM are naturally rarer and occur in narrower and more specialised habitats (category H). Furthermore, the most common birds are generally smaller and occur in a broader range of habitats (category A) (particularly if ostriches are excluded as an outlier). And, while penguins are rarely used and probably opportunistically acquired in Africa, the specific trade in many Accipitridae, owls, large hornbills (particularly Abyssinian and Southern Ground-hornbills) and, in such countries as Cote d'Ivoire, heads of Black-crowned Crane (*Balearica pavonina*), is cause for concern (Tables 3 & 9). Furthermore, vulnerable nest types and sites, combined with small clutch sizes may be a compounding factor in the vulnerability of species to selective harvesting.

It is widely accepted, for example, that birds using closed nests (such as natural or excavated cavities in trees) are prone to lower levels of natural nest predation [89]. However, adults using closed nests may be more easily trapped/killed as the sites are often re-used and accordingly the species are more vulnerable to detection and predation by people. Nearly half of the 19 most-frequently traded bird species recorded in African TM markets (hoopoes, hornbills, parrots, rollers and owls), nest in tree cavities (Table 4). While human predation on hole- and open-nesting bird species is a conservation challenge, the behaviour of some species also offers an opportunity for harvest and rearing for TM of redundant second-hatched chicks normally starved or killed by the first-hatched elder sibling [90], and provision of safe artificial nests [91]

In addition to threats from the African TM trade, many families are under selective pressure from anthropogenic factors such as collisions with aircraft and overhead power lines and, most importantly, poisoning. The Accipitridae, especially vultures, are frequently victims of these threats [78], [79], and large-scale mortalities have been caused by deliberate and inadvertent poisoning with such pesticides as the carbamate-based pesticide Furadan [92]–[94]. In South Africa, poisoning has been the main factor causing a decline in Bateleur Eagle [95] and maybe Tawny Eagle (*Aquila rapax*) and Southern Ground-hornbill populations [96]. In Kenya, pesticides have been implicated in the decline of several species and populations, including raptors and Mackinder's Eagle-owls [72], [88], [94].

Effective and appropriate conservation strategies for utilised species should ideally be based on a thorough understanding of the world views of the users, such as why particular birds are selected across diverse African healing traditions, together with a thorough understanding of the species' biology and the TM supply chains for those species. An appreciation of the cultural and religious context is essential to develop viable conservation strategies. Selection of particular groups of birds is based on similar traits or combinations of characteristics that have symbolic meaning. Examples of these factors are the large body size and red, black and white colours of both ground-hornbill species and Bateleur eagles. In Nigeria, the raucous calls of Western Grey Plantain-eaters (*Crinifer piscator*) and three turaco species (Violet, Guinea and Yellow-billed) (*Musophaga violacea, Tauraco persa, T. macrorhynchus*) are believed to attract customers, the melodious calls of the Black-crowned Tchagra (*Tchagra senegalensis*) to impart musical ability [17]. It is well known from distributions of bird species that African avifauna vary from country to country, so it is not surprising that different species within the same groups of birds sharing common characteristics are used. Across Africa, however, birds are ritually potent symbols that have metaphorical meaning in healing and religious traditions that link mind and body, and this potency connects to widespread beliefs in the power of wild places and the meaning of natural events. In the Congo, for example, 46 bird species were documented that Mbuti hunter-gatherers believe are mediators between the spirit world and human society, and 20 bird species with the power to cause illness [97]. It is a mistake to think that Mbuti beliefs are archaic and isolated, since our work shows that belief in the ritual potency of birds is widespread. It is not by chance that the Great Blue Turacos (*Corythaeola cristata*), Senegal Coucals (*Centropus senegalensis*) and egrets, that the Mbuti consider dangerously powerful, are also sold in urban markets. The eerie calls of nocturnal birds such as owls are a further example, with widespread symbolic beliefs attributed to a range of owl species [3], [88], [98]. The similarity of use of avifaunal orders is high, especially at a morphospecies level (Table 5), suggesting an opportunity for a common understanding of what drives demand across the continent. This also suggests common links across African belief systems, and offers an opportunity for similar conservation and resource-management strategies across a wide range of countries that recognise cultural links to African bird diversity.

## Conclusions

Bird conservation policies and practices in Africa need to take into account bird use for traditional purposes, particularly in national and regional conservation strategies across West and southern Africa. The selection of taxa for TM involves layers of anthropological, ecological, behavioural and phenotypic complexity. Across Africa, large and/or conspicuous birds are targeted by the TM trade. Since bigger birds tend to occur at lower population densities and have slower reproductive rates, which makes them rarer and with lower population numbers per unit area of suitable habitat, their populations are more vulnerable, especially if their habitats are also being transformed. Vultures and hornbills, and such other large avifauna as eagles and bustards, are the most vulnerable taxa. Selective harvesting of these vulnerable birds for the TM trade can no longer be ignored. Bird conservation strategies need to take the TM trade into account, starting with the 19 species listed in this study (Table 9).

It must be noted that the TM trade in wild birds is only a part, probably a relatively small one, of the commercial trade in wild birds in Africa. Other important forms of trade include birds hunted primarily for food ('bush meat') and, probably most voluminous of all, trade in live birds for aviculture and pets. Each form of trade is probably to an extent integrated and interdependent, since all involve initially hunters and finally traders. For example, food items might end up in TM if not consumed while fresh, and pre-export casualties from the live-bird trade are potentially available as food while fresh and later for TM. The different forms and proportions of each trade type probably vary by bird taxa and country, for example with bush meat trade expected for larger birds in forested areas and live-bird trade best known for parrots and finches and from Tanzania and Senegal. It would be revealing, therefore, to have similar analyses of use by species and country conducted for these non-TM forms of trade, so that a comprehensive understanding can be established of how these different trades integrate into their combined effect on avifaunal conservation.

## Supporting Information

Figure S1 **The mean mass (g) of species sold in ZA, NG and BJ markets, and the total for seven countries combined (‘All traded’), in each Rabinowitz category of commonness and rarity (excluding ostrich).** Birds from ZA tended to be larger and were almost twice as heavy as birds from BJ and NG.
(TIFF)

Table S1 Inventory of avian species used and sold for traditional medicine in 25 African countries with their IUCN conservation statuses, Rabinowitz rarity classification, population trend, and the number of countries in which they were recorded to be sold in markets. All abbreviations are listed as footnotes at the end of the table. The taxonomic classification follows BirdLife.
(DOC)

Tables S2 **a,b,c.** Conservation priority groupings, and hence vulnerability to exploitation, of species sold in traditional medicine markets across Africa. Species were sorted into two groups of higher and lower priority and vulnerability according to the K-means cluster analysis. Species are listed within the groups in descending order of their maximum scores for the three standardised variables.
(DOC)

Table S3 Mean EOOs for traded bird species in the eight Rabinowitz and four rarity categories (corresponding with the classes in Table 1), where A and 4 are the most common species, and H and 1 are most rare species (excludes PALs and exotics). Birds with an EOO >6,790,00 km^2^ were assigned to classes A to D.
(DOCX)

Table S4 Comparison between the number of species per order recorded as being used for traditional medicine in Africa (this study), Brazil (from [9], [10], [75], [76]) and India (from [11], [78]–[80]). Taxa arranged according to the orders common.
(DOCX)
