# Supplementary figures and images for: Risks to Birds Traded for African Traditional Medicine: A Quantitative Assessment
Source: PLoS One. 2014 Aug 27;9(8):e105397. doi: 10.1371/journal.pone.0105397 (PMC4146541; doi:10.1371/journal.pone.0105397)

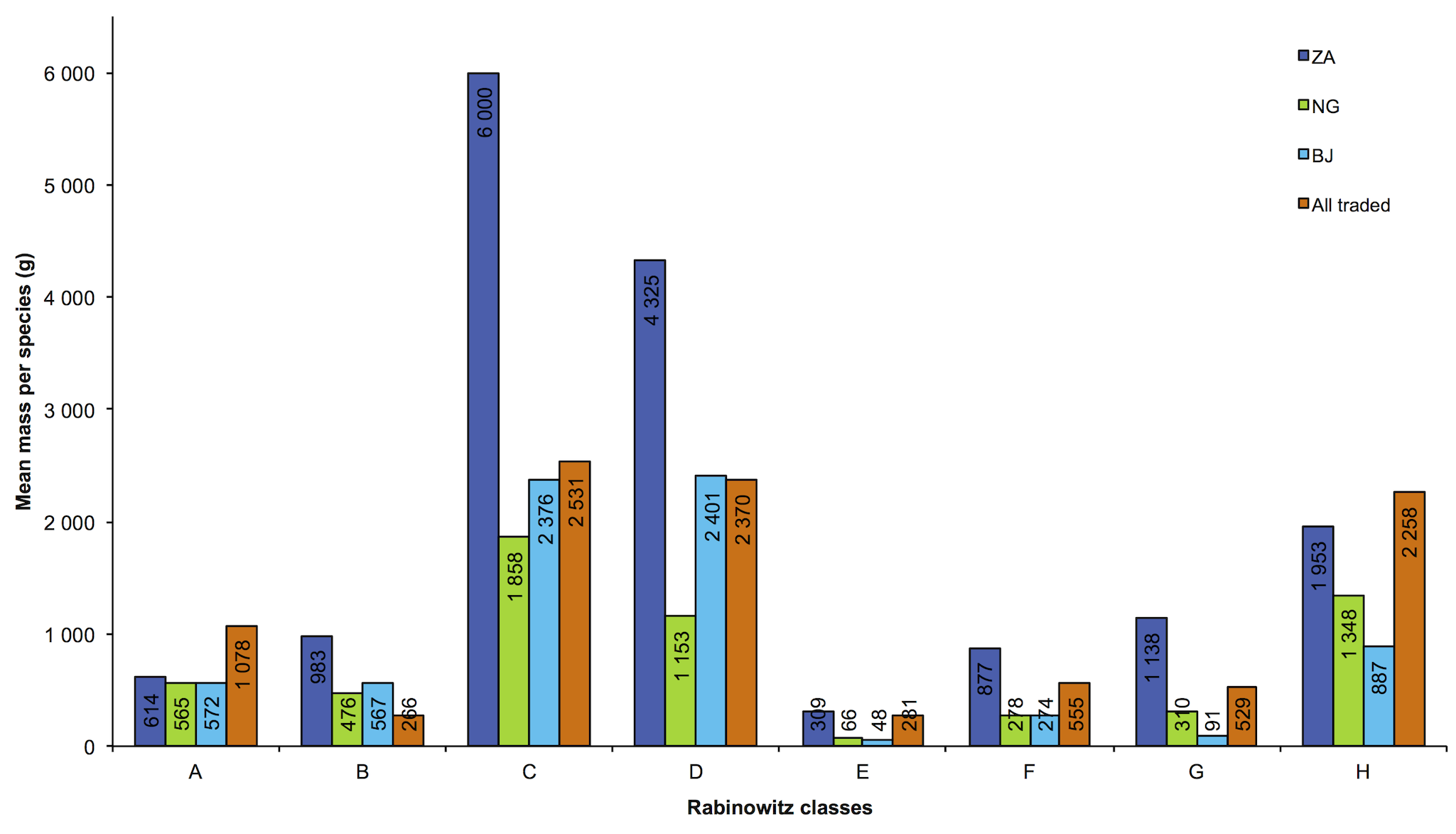

Supplement: Figure S1 — The mean mass (g) of species sold in ZA, NG and BJ markets, and the total for seven countries combined (‘All traded’), in each Rabinowitz category of commonness and rarity (excluding ostrich). Birds from ZA tended to be larger and were almost twice as heavy as birds from BJ and NG. (TIFF) [file pone.0105397.s001.tiff]
