# Supplementary material for: Risks to Birds Traded for African Traditional Medicine: A Quantitative Assessment
Source: PLoS One. 2014 Aug 27;9(8):e105397. doi: 10.1371/journal.pone.0105397 (PMC4146541; doi:10.1371/journal.pone.0105397)
Supplement: Table S1 — Inventory of avian species used and sold for traditional medicine in 25 African countries with their IUCN conservation statuses, Rabinowitz rarity classification, population trend, and the number of countries in which they were recorded to be sold in markets. All abbreviations are listed as footnotes at the end of the table. The taxonomic classification follows BirdLife. (DOC) [file pone.0105397.s002.doc]

**Supporting Information Table S1. Species inventory and checklist of avian species found to be used and/or traded for traditional medicine in 25 African countries.** The classification and nomenclature follows BirdLife International (correct to June 2014).

| Classification  (**ORDER**, Family) | Species | Common name | IUCN category ^a^ | Rarity classification ^b^ | Population trend ^c^ | Countries where use and traded are reported |
| --- | --- | --- | --- | --- | --- | --- |
| **ANSERIFORMES** | |  |  |  |  |  |
| Anatidae | *Anas acuta*^PAL^ | Pintail, Northern | LC | B | D | NG |
|  | *Anas undulata* | Duck, Yellow-billed | LC | B | S | NG |
|  | *Dendrocygna bicolor* | Whistling-duck, Fulvous | LC | B | D | NG,SD |
|  | *Dendrocygna viduata* | Whistling-duck, White-faced | LC | B | I | BJ,NG,ZA |
|  | *Plectropterus gambensis* | Goose, Spur-winged | LC | A | I | BJ,NG |
|  | *Pteronetta hartlaubii* | Duck, Hartlaub's | LC | F | D | NG |
|  | *Thalassornis leuconotus* | Duck, White-backed | LC | D | D | ZA |
| - | - | Duck, unidentified | - | - | - | NA,TG,ZA |
| **APODIFORMES** | |  |  |  |  |  |
| Apodidae | *Apus affinis* | Swift, Little | LC | A | I | NG |
|  | *Apus caffer* | Swift, White-rumped | LC | B | I | NG |
| **BUCEROTIFORMES** | |  |  |  |  |  |
| Bucerotidae | *Bycanistes bucinator* | Hornbill, Trumpeter | LC | F | S | ZA |
|  | *Bycanistes cylindricus* | Hornbill, Brown-cheeked | VU | H | D | BJ,NG |
|  | *Bycanistes fistulator* | Hornbill, Piping | LC | F | S | CI,NG,ZA |
|  | *Bycanistes subcylindricus* | Hornbill, Black-and-white-casqued | LC | F | ? | BJ,CD,NG |
|  | *Ceratogymna atrata* | Hornbill, Black-casqued | LC | F | D | BJ,CD,NG |
|  | *Ceratogymna elata* | Hornbill, Yellow-casqued | VU | H | D | NG |
|  | *Tockus camurus* | Hornbill, Red-billed Dwarf | LC | F | S | NG |
|  | *Tockus erythrorhynchus* | Hornbill, Red-billed | LC | B | S | BF,BJ,NG,ZA |
|  | *Tockus fasciatus* | Hornbill, African Pied | LC | F | ? | BF,BJ,CI,NG,TG |
|  | *Tockus hartlaubi* | Hornbill, Black Dwarf | LC | F | S | NG |
|  | *Tockus nasutus* | Hornbill, African Grey | LC | A | S | BF,BJ,NG |
|  | *Tockus* sp. | Hornbill, unidentified | - | - | - | CM,SD,TG,ZA |
|  | *Tropicranus albocristatus* | Hornbill, White-crested | LC | F | S | CD,NG |
| Bucorvidae | *Bucorvus abyssinicus* | Ground-hornbill, Abyssinian | LC | H | S | BF,BJ,ET,ML,NG |
|  | *Bucorvus leadbeateri* | Ground-hornbill, Southern | VU | D | D | KE,NA,SD,ZA,ZW |
| **Caprimulgiformes** | |  |  |  |  |  |
| Caprimulgidae | *Caprimulgus climacurus* | Nightjar, Long-tailed | LC | C | S | BJ,NG |
|  | *Caprimulgus nigriscapularis* | Nightjar, Black-shouldered | LC | F | S | NG |
|  | *Caprimulgus ruficollis*^PAL^ | Nightjar, Red-necked | LC | H | D | MA |
|  | *Caprimulgus* sp. | Nightjar, unidentified | - | - | - | BJ,ZA |
|  | *Macrodipteryx longipennis* | Nightjar, Standard-winged | LC | E | S | BJ,BG |
|  | *Macrodipteryx vexillarius* | Nightjar, Pennant-winged | LC | G | S | MW |
| **CHARADIIFORMES** | |  |  |  |  |  |
| Burhinidae | *Burhinus capensis* | Thick-knee, Spotted | LC | A | S | NG,ZA |
|  | *Burhinus senegalensis* | Thick-knee, Senegal | LC | C | ? | BF,NG |
|  | *Burhinus* sp*.* | Thick-knee, unidentified | - | - | - | ZA |
| Charadriidae | *Charadrius alexandrinus*^PAL^ | Plover, Kentish | LC | B | D | BJ |
|  | *Vanellus albiceps* | Lapwing, White-headed | LC | B | S | BJ |
|  | *Vanellus armatus* | Lapwing, Blacksmith | LC | F | I | ZA |
|  | *Vanellus coronatus* | Lapwing, Crowned | LC | B | I | ZA |
|  | *Vanellus crassirostris* | Lapwing, Long-toed | LC | F | ? | NG |
|  | *Vanellus lugubris* | Lapwing, Senegal | LC | H | ? | BJ,CD,NG,ZA |
|  | *Vanellus senegallus* | Lapwing, Wattled | LC | B | S | NG |
|  | *Vanellus tectus* | Lapwing, Black-headed | LC | F | ? | BF,NG |
| Glareolidae | *Glareola pratincola* | Pratincole, Collared | LC | B | D | BJ |
|  | *Pluvianus aegyptius* | Plover, Egyptian | LC | H | D | BJ |
|  | *Rhinoptilus chalcopterus* | Courser, Bronze-winged | LC | A | S | NG |
| Jacanidae | *Actophilornis africanus* | Jacana, African | LC | B | S | BJ |
| Laridae | *Larus cirrocephalus* | Gull, Grey-headed | LC | B | S | ZA |
|  | *Larus* sp. | Gull, unidentified | - | - | - | ZA |
|  | *Sterna caspia* | Tern, Caspian | LC | B | I | ZA |
| Recurvirostridae | *Recurvirostra avosetta* | Avocet, Pied | LC | B | ? | ZA |
| Scolopacidae | *Gallinago media*^PAL^ | Snipe, Great | NT | G | D | BJ |
|  | *Lymnocryptes minimus*^PAL^ | Snipe, Jack | LC | C | S | BJ |
| **Ciconiiformes** | |  |  |  |  |  |
| Ardeidae | *Ardea cinerea* | Heron, Grey | LC | A | ? | NG,TG,ZA |
|  | *Ardea goliath* | Heron, Goliath | LC | D | S | BJ,CI |
|  | *Ardea melanocephala* | Heron, Black-headed | LC | A | I | CD,NG |
|  | *Ardea purpurea* | Heron, Purple | LC | B | D | BJ |
|  | *Ardea* sp. | Heron, unidentified | - | - | - | BF,BJ |
|  | *Ardeola ralloides* | Heron, Squacco | LC | B | D | NG |
|  | *Bubulcus ibis* | Egret, Cattle | LC | A | I | BF,BJ,NG,ZA |
|  | *Butorides striata* | Heron, Striated | LC | A | D | BJ,NG |
|  | *Casmerodius albus* | Egret, Great | LC | A | ? | ZA |
|  | *Egretta garzetta* | Egret, Little | LC | A | I | BJ |
|  | *Egretta gularis* | Reef-egret, Western | LC | H | S | TG |
|  | *Egretta* sp. | Egret, unidentified | - | - | - | BF |
|  | *Gorsachius leuconotus* | Night-heron, White-backed | LC | D | S | NG |
|  | *Ixobrychus sturmii* | Bittern, Dwarf | LC | D | ? | NG |
|  | *Mesophoyx intermedia* | Egret, Intermediate | LC | A | D | NG |
|  | *Nycticorax nycticorax* | Night-heron, Black-crowned | LC | B | D | BJ,NG,TG,ZA |
|  | *Tigriornis leucolopha* | Tiger-heron, White-crested | LC | H | ? | NG |
|  | - | Egret, unidentified | - | - | - | BF,TG |
|  | - | Night-Heron, unidentified | - | - | - | TG |
| Ciconiidae | *Anastomus lamelligerus* | Openbill, African | LC | B | D | NG |
|  | *Ciconia abdimii* | Stork, Abdim's | LC | B | D | NG |
|  | *Ciconia ciconia*^PAL^ | Stork, White | LC | B | I | ZA |
|  | *Leptoptilos crumeniferus* | Stork, Marabou | LC | A | I | BJ,NG |
|  | *Mycteria ibis* | Stork, Yellow-billed | LC | B | D | BF |
|  | - | Stork, unidentified | - | - | - | ZA |
| **COLIIFORMES** | |  |  |  |  |  |
| Coliidae | *Colius striatus* | Mousebird, Speckled | LC | A | I | CD,ZA |
|  | - | Mousebird, unidentified | - | - | - | ZA |
| **COLUMBIIFORMES** | |  |  |  |  |  |
| Columbidae | *Columba arquatrix* | Olive-pigeon, African | LC | F | D | CD |
|  | *Columba guinea* | Pigeon, Speckled | LC | A | S | NG,ZA |
|  | *Columba livia* | Pigeon, Rock | LC | A | D | ZA |
|  | *Columba uncinata* | Pigeon, Afep | LC | F | S | BJ |
|  | *Columba* sp. | Pigeon, unidentified | - | - | - | BJ,NG |
|  | *Stigmatopelia senegalensis* | Dove, Laughing | LC | A | S | ZA |
|  | *Streptopelia capicola* | Dove, Ring-necked | LC | A | I | NG,ZA |
|  | *Streptopelia semitorquata* | Dove, Red-eyed | LC | A | I | BJ,CD,NG,ZA |
|  | *Streptopelia* sp. | Dove, unidentified | - | - | - | ET,ZW |
|  | *Treron calvus* | Green-pigeon, African | LC | A | D | BJ |
|  | *Treron waalia* | Green-pigeon, Bruce's | LC | E | D | NG |
|  | *Turtur afer* | Wood-dove, Blue-spotted | LC | A | S | CD,NG |
|  | - | Dove, unidentified | - | - | - | ET,TG,ZA,ZW |
| **CORACIIFORMES** | |  |  |  |  |  |
| Alcedinidae | *Alcedo cristata* | Kingfisher, Malachite | LC | B | S | BJ,CD,NG,ZA |
|  | *Ceryle rudis* | Kingfisher, Pied | LC | B | ? | BJ,ZA |
|  | *Ceyx pictus* | Pygmy-kingfisher, African | LC | A | S | BJ,NG |
|  | *Halcyon albiventris* | Kingfisher, Brown-hooded | LC | E | S | ZA |
|  | *Halcyon chelicuti* | Kingfisher, Striped | LC | A | S | NG |
|  | *Halcyon leucocephala* | Kingfisher, Grey-headed | LC | A | S | BJ,CD,NG |
|  | *Halcyon malimbica* | Kingfisher, Blue-breasted | LC | E | D | NG |
|  | *Halcyon senegalensis* | Kingfisher, Woodland | LC | A | S | BJ,CI,NG |
|  | *Megaceryle maxima* | Kingfisher, Giant | LC | B | D | NG,ZA |
|  | - | Kingfisher, unidentified | - | - | - | BF,ZA,ZW |
| Coraciidae | *Coracias abyssinicus* | Roller, Abyssinian | LC | A | I | BF,BJ,GH,NG,TG |
|  | *Coracias cyanogaster* | Roller, Blue-bellied | LC | H | D | BJ,NG |
|  | *Coracias garrulus*^PAL^ | Roller, European | NT^f^ | A | D | BF,BJ,NG |
|  | *Coracias naevia* | Roller, Rufus-crowned | LC | C | D | BF,NG |
|  | *Eurystomis glaucurus* | Roller, Broad-billed | LC | A | S | BJ |
|  | *Eurystomis gularis* | Roller, Blue-throated | LC | F | D | CD |
|  | *Eurystomis* sp. | Roller, unidentified | - | - | - | BJ,NG |
| Meropidae | *Merops albicollis* | Bee-eater, White-throated | LC | F | S | BJ,NG |
|  | *Merops malimbicus* | Bee-eater, Rosy | LC | F | ? | NG |
|  | *Merops nubicus* | Bee-eater, Northern Carmine | LC | F | D | BJ,NG |
|  | *Merops oreobates* | Bee-eater, Cinnamon-chested | LC | F | ? | CD |
|  | *Merops pusillus* | Bee-eater, Little | LC | A | D | NG |
| Phoeniculidae | *Phoeniculus casteneiceps* | Woodhoopoe, Forest | LC | H | D | NG |
|  | *Phoeniculus purpureus* | Woodhoopoe, Green | LC | B | D | BJ,NG |
|  | *Phoeniculus* sp*.* | Woodhoopoe, unidentified | - | - | - | ZA |
|  | *Rhinopomastus aterrimus* | Scimitarbill, Black | LC | B | D | BJ |
| Upupidae | *Upupa epops* | Hoopoe, Eurasian | LC | A | D | BF,MA,NG,SD,ZA |
| **CUCULIFORMES** | |  |  |  |  |  |
| Cuculidae | *Centropus grillii* | Coucal, Black | LC | D | S | BJ,NG |
|  | *Centropus leucogaster* | Coucal, Black-throated | LC | F | S | BJ,NG |
|  | *Centropus monachus* | Coucal, Blue-headed | LC | E | S | CD |
|  | *Centropus senegalensis* | Coucal, Senegal | LC | C | S | BF,BJ,NG |
|  | *Centropus superciliosus* | Coucal, White-browed | LC | B | S | BJ,ZA |
|  | *Ceuthmochares aereus* | Yellowbill | LC | H | S | BJ,CD,NG,ZA |
|  | *Chrysococcyx caprius* | Cuckoo, Didric | LC | A | S | BJ,NG |
|  | *Clamator glandarius* | Cuckoo, Great Spotted | LC | A | S | NG |
|  | *Clamator jacobinus* | Cuckoo, Pied | LC | A | S | NG |
|  | *Clamator levaillantii* | Cuckoo, Levaillant's | LC | E | S | BJ,NG |
|  | *Cuculus canorus*^PAL^ | Cuckoo, Common | LC | A | D | CD |
|  | *Cuculus clamosus* | Cuckoo, Black | LC | A | S | NG |
|  | *Cuculus gularis* | Cuckoo, African | LC | A | S | BJ,NG |
| **FALCONIFORMES** | |  |  |  |  |  |
| Accipitridae | *Accipiter badius* | Shikra | LC | A | S | BJ,CD,NG |
|  | *Accipiter erythropus* | Sparrowhawk, Red-thighed | LC | H | D | NG |
|  | *Accipiter tachiro* | Goshawk, African | LC | B | D | NG |
|  | *Aquila rapax* | Eagle, Tawny | LC | A | S | NG,ZA |
|  | *Aquila wahlbergi* | Eagle, Wahlberg's | LC | A | S | NG |
|  | *Aviceda cuculoides* | African Baza | LC | D | S | NG |
|  | *Butastur rufipennis* | Buzzard, Grasshopper | LC | A | D | NG |
|  | *Buteo auguralis* | Buzzard, Red-necked | LC | A | I | BJ,NG |
|  | *Buteo rufofuscus* | Buzzard, Jackal | LC | E | S | ZA |
|  | *Chelictinia riocourii* | Kite, African Swallow-tailed | LC | F | D | NG |
|  | *Circaetus beaudouini* | Snake-eagle, Beaudouin's | VU | H | D | NG |
|  | *Circaetus cinerascens* | Snake-eagle, Banded | LC | C | D | NG |
|  | *Circaetus cinereus* | Snake-eagle, Brown | LC | C | S | NG |
|  | *Circus aeruginosus* | Marsh-harrier, Western | LC | B | I | BJ |
|  | *Circus macrourus*^PAL^ | Harrier, Pallid | NT | D | D | BJ |
|  | *Circus pygargus*^PAL^ | Harrier, Montagu's | LC | C | D | BJ |
|  | *Dryotriorchis spectabilis* | Serpent-eagle, Congo | LC | H | D | NG |
|  | *Elanus caeruleus* | Kite, Black-winged | LC | A | S | BJ,NG,ZA |
|  | *Gypaetus barbatus* | Lammergeier | LC | D | D | ZA |
|  | *Gypohierax angolensis* | Vulture, Palm-nut | LC | B | S | NG |
|  | *Gyps africanus* | Vulture, White-backed | EN | C | D | BJ,NG,ZA |
|  | *Gyps coprotheres* | Vulture, Cape | VU | H | D | LS,MZ,ZA |
|  | *Gyps fulvus* | Vulture, Griffon | LC | C | I | MA |
|  | *Gyps rueppellii* | Vulture, Rueppell's | EN | C | D | BJ,CI,ML,NG |
|  | *Haliaeetus vocifer* | Fish-eagle, African | LC | B | S | BJ,NG,ZA,ZW |
|  | *Kaupifalco monogrammicus* | Buzzard, Lizard | LC | A | S | BJ,NG |
|  | *Lophaetus occipitalis* | Eagle, Long-crested | LC | B | I | BJ,CD,NG |
|  | *Melierax gabar* | Goshawk, Gabar | LC | B | S | NG |
|  | *Melierax metabates* | Chanting-Goshawk, Dark | LC | B | S | NG |
|  | *Milvus migrans* | Kite, Black | LC | A | D | BJ,NG |
|  | *Milvus* sp. | Kite, unidentified | - | - | - | BF |
|  | *Necrosyrtes monachus* | Vulture, Hooded | EN | A | D | BJ,CI,GH,NG,TG,ZA |
|  | *Neophron percnopterus* | Vulture, Egyptian | EN | C | D | BJ,NG |
|  | *Polemaetus bellicosus* | Eagle, Martial | VU | C | D | CM |
|  | *Polyboroides typus* | Harrier-hawk, African | LC | A | S | NG,TG,ZA |
|  | *Sagittarius serpentarius* | Secretarybird | VU | D | D | ZA |
|  | *Stephanoaetus coronatus* | Hawk-eagle, Crowned | NT | H | D | CM,NG.ZA |
|  | *Terathopius ecaudatus* | Bateleur | NT | B | D | NG,ZA |
|  | *Torgos tracheliotos* | Vulture, Lappet-faced | VU | D | D | BJ,ZA,ZM |
|  | *Trigonoceps occipitalis* | Vulture, White-headed | VU | D | D | CD,MZ,NG,ZA |
|  | - | Eagle, unidentified | - | - | - | BF,CM,ET,KE,TG,ZA,ZW |
|  | - | Hawk, unidentified | - | - | - | BF,BJ,TG |
|  | - | Vulture, unidentified. | - | - | - | BW,MZ,SZ,TG,ZA,ZM,ZW |
| Falconidae | *Falco alopex* | Kestrel, Fox | LC | H | S | BJ |
|  | *Falco ardosiaceus* | Kestrel, Grey | LC | B | S | BJ,NG |
|  | *Falco biarmicus* | Falcon, Lanner | LC | A | I | MA,NG |
|  | *Falco chicquera* | Falcon, Red-necked | LC | D | S | NG |
|  | *Falco cuvierii* | Hobby, African | LC | D | D | NG |
|  | *Falco tinnunculus* | Kestrel, Common | LC | A | D | BJ,NG |
|  | *Falco* sp. | Kestrel, unidentified | - | - | - | ZA |
| **GALLIFORMES** | |  |  |  |  |  |
| Numididae | *Guttera plumifera* | Guineafowl, Plumed | LC | F | D | CD |
|  | *Guttera pucherani* | Guineafowl, Crested | LC | F | S | BJ,NG,ZA |
|  | *Numida meleagris* | Guineafowl, Helmeted | LC | A | S | BF,BJ,ET,NG,ZA,ZW |
|  | - | Guineafowl, unidentified | - | - | - | CM,MZ,NG |
| Phasianidae | *Afropavo congensis* | Peafowl, Congo | VU | H | D | CD |
|  | *Coturnix chinensis* | Quail, Blue | LC | D | S | BJ |
|  | *Coturnix coturnix* | Quail, Common | LC | B | D | NG,ZA |
|  | *Coturnix delegorguei* | Quail, Harlequin | LC | B | S | BJ,NG |
|  | *Coturnix* sp. | Quail, unidentified | - | - | - | CM |
|  | *Francolinus afer* | Spurfowl, Red-necked | LC | E | D | CD |
|  | *Francolinus ahantensis* | Francolin, Ahanta | LC | F | D | BJ,NG |
|  | *Francolinus albogularis* | Francolin, White-throated | LC | H | S | BF |
|  | *Francolinus bicalcaratus* | Fancolin, Double-spurred | LC | F | D | BF,NG |
|  | *Francolinus* sp. | Francolin, unidentified | - | - | - | BF,BJ,CI,NG,TG,ZA |
|  | *Pavo cristatus* | Peafowl, Indian^e^ | LC | H | S | NG,ZA |
|  | *Ptilopachus petrosus* | Partridge, Stone | LC | F | S | NG |
| **GRUIFORMES** | |  |  |  |  |  |
| Gruidae | *Balearica pavonina* | Crowned-crane, Black | VU | H | D | BJ,CI,NG |
| Heliornithidae | *Podica senegalensis* | Finfoot, African | LC | D | D | NG |
| Otididae | *Ardeotis arabs* | Bustard, Arabian | NT | H | D | CI,NG |
|  | *Ardeotis kori* | Bustard, Kori | NT | H | D | NA |
|  | *Eupodotis melanogaster* | Bustard, Black-bellied | LC | D | D | NG |
|  | *Eupodotis savilei* | Bustard, Savile's | LC | G | S | CI |
|  | *Eupodotis senegalensis* | Bustard, White-bellied | LC | G | D | NG |
|  | *Eupodotis* sp. | Bustard, unidentified | - | - | - | BF |
|  | *Neotis denhami* | Bustard, Denham's | NT | D | D | NG |
|  | - | Bustard, unidentified | - | - | - | NA |
| Rallidae | *Amaurornis flavirostra* | Crake, Black | LC | B | ? | BJ,NG,ZA |
|  | *Canirallus oculeus* | Rail, Grey-throated | LC | H | D | BJ |
|  | *Crecopsis egregia* | Crake, African | LC | C | S | NG |
|  | *Gallinula angulata* | Moorhen, Lesser | LC | B | ? | BJ,NG |
|  | *Gallinula chloropus* | Moorhen, Common | LC | F | ? | NG,ZA |
|  | *Gallinula* sp. | Moorhen, unidentified | - | - | - | ZA |
|  | *Himantornis haematopus* | Rail, Nkulengu | LC | F | D | NG |
|  | *Porphyrio alleni* | Gallinule, Allen's | LC | B | D | BJ |
|  | *Porphyrio porphyrio* | Swamphen, Purple | LC | B | ? | BJ,NG,ZA |
|  | *Sarothrura pulchra* | Flufftail, White-spotted | LC | F | D | BJ |
| **MUSOPHAGIFORMES** | |  |  |  |  |  |
| Musophagidae | *Corythaeola cristata* | Turaco, Great Blue | LC | F | S | BJ,CD,NG |
|  | *Crinifer piscator* | Plantain-eater, Western Grey | LC | F | S | BF,BJ,NG,TG |
|  | *Musophaga rossae* | Turaco, Ross's | LC | F | S | CD |
|  | *Musophaga violacea* | Turaco, Violet | LC | F | S | BJ,NG |
|  | *Tauraco corythaix* | Turaco, Knysna | LC | H | S | SZ,ZA |
|  | *Tauraco macrorhynchus* | Turaco, Yellow-billed | LC | F | S | NG |
|  | *Tauraco persa* | Turaco, Guinea | LC | F | S | BJ,CM,NG |
|  | *Tauraco porphyreolophus* | Turaco, Purple-crested | LC | F | D | ZA |
|  | - | Turaco, unidentified | - | - | - | CM |
| **PASSERIFORMES** | |  |  |  |  |  |
| Campephagidae | *Coracina pectoralis* | Cuckooshrike, White-breasted | LC | F | D | NG |
| Cisticolidae | *Apalis porphyrolaema* | Apalis, Chestnut-throated | LC | F | S | CD |
|  | *Cisticola erythrops* | Cisticola, Red-faced | LC | F | S | CD |
|  | *Cisticola* sp | Cisticola, unidentified | - | - | - | ZA |
|  | *Hypergerus atriceps* | Warbler, Oriole | LC | F | S | NG |
|  | *Prinia bairdii* | Prinia, Banded | LC | E | S | CD |
|  | *Prinia subflava* | Prinia, Tawny-flanked | LC | A | S | ZA |
| Corvidae | *Corvus albicollis* | Raven, White-necked | LC | E | D | ZA |
|  | *Corvus albus* | Crow, Pied | LC | A | S | BF,BH,CD,ET,NG,TG,ZA,ZW |
|  | *Corvus capensis* | Crow, Cape | LC | E | I | ZA |
|  | *Corvus rhipidurus* | Raven, Fan-tailed | LC | G | D | ET |
|  | *Corvus* sp*.* | Crow, unidentified | - | - | - | CM,ET,NA,ZA |
|  | *Ptilostomus afer* | Piapiac | LC | E | S | NG |
| Dicruridae | *Dicrurus adsimilis* | Drongo, Fork-tailed | LC | A | S | BJ,NG |
| Estrildidae | *Cryptospiza salvadorii* | Crimson-wing, Abyssinian | LC | F | S | CD |
|  | *Estrilda astrild* | Waxbill, Common | LC | E | S | CD |
|  | *Estrilda atricapilla* | Waxbill, Black-headed | LC | E | S | CD |
|  | *Estrilda trogolodytes* | Waxbill, Black-rumped | LC | F | S | NG |
|  | *Lagonostica senegala* | Firefinch, Red-billed | LC | A | S | BJ,NG |
|  | *Lonchura bicolor* | Munia, Black-and-white | LC | E | S | CD |
|  | *Lonchura cucullata* | Munia, Bronze | LC | A | S | CD |
|  | *Nesocharis ansorgei* | Oliveback, White-collared | LC | F | S | CD |
|  | *Nigrita canicapillus* | Negrofinch, Grey-headed | LC | F | S | CD |
|  | *Spermophaga haematina* | Bluebill, Western | LC | F | S | NG |
|  | *Spermophaga ruficapilla* | Bluebill, Red-headed | LC | E | S | CD |
| Fringillidae | *Serinus sulphuratus* | Canary, Brimstone | LC | E | S | CD |
| Hirundinidae | *Delichon urbicum*^PAL^ | House-martin, Northern | LC | A | D | NG |
|  | *Hirundo abyssinica* | Striped-swallow, Lesser | LC | A | I | BJ |
|  | *Hirundo aethiopica* | Swallow, Ethiopian | LC | E | I | BJ,NG |
|  | *Hirundo angolensis* | Swallow, Angola | LC | H | I | CD |
|  | *Hirundo rustica*^PAL^ | Swallow, Barn | LC | A | D | BJ,NG |
|  | *Hirundo smithii* | Swallow, Wire-tailed | LC | B | I | BJ,CD |
|  | *Hirundo* sp*.* | Swallow, unidentified | - | - | - | ZA,ZM |
|  | *Psalidoprocne nitens* | Saw-wing, Square-tailed | LC | E | D | CD |
|  | *-* | Swallow, unidentified | - | - | - | NG |
| Laniidae | *Corvinella corvina* | Shrike, Yellow-billed | LC | F | ? | BJ,NG |
|  | *Lanius collaris* | Fiscal, Common | LC | A | I | BJ,ZA |
| Malaconotidae | *Dryoscopus gambensis* | Puffback, Northern | LC | F | S | BJ |
|  | *Laniarius aethiopicus* | Boubou, Ethiopian | LC | E | S | CD,NG |
|  | *Laniarius barbarus* | Gonolek, Common | LC | E | S | NG |
|  | *Laniarius ferrugineus* | Boubou, Southern | LC | E | S | ZA |
|  | *Malaconotus blanchoti* | Bush-shrike, Grey-headed | LC | B | I | BJ,NG |
|  | *Prionops plumatus* | Helmet-Shrike, White | LC | A | S | BJ,NG,ZA |
|  | *Tchagra senegalus* | Tchagra, Black-crowned | LC | A | S | BF,BJ,NG |
|  | *Telephorus multicolor* | Bush-shrike, Many-coloured | LC | F | S | BJ |
| Monarchidae | *Terpsiphone rufiventer* | Paradise-Flycatcher, Black-headed | LC | F | D | BJ,CD |
|  | *Terpsiphone viridis* | Paradise-Flycatcher, African | LC | B | S | BJ |
| Motacillidae | *Macronyx* sp*.* | Longclaw, unidentified | - | - | - | ZA |
|  | *Motacilla aguimp* | Wagtail, African Pied | LC | B | S | CD,NG |
|  | *Motacilla capensis* | Wagtail, Cape | LC | F | S | CD |
|  | *Motacilla flava*^PAL^ | Wagtail, Yellow | LC | B | D | BJ |
| Muscicapidae | *Cossypha albicapilla* | Robin-chat, White-crowned | LC | H | S | BJ,NG |
|  | *Cossypha natalensis* | Robin-chat, Red-capped | LC | F | S | ZA |
|  | *Cossypha niveicapilla* | Robin-chat, Snowy-crowned | LC | F | S | NG |
|  | *Cossypha* sp*.* | Robin-chat, unidentified | - | - | - | BJ,CI |
|  | *Melaenornis edolioides* | Flycatcher, Northern Black | LC | E | S | NG |
|  | *Melaenornis pammelaina* | Flycatcher, Southern Black | LC | E | S | ZA |
|  | *Muscicapa striata*^PAL^ | Flycatcher, Spotted | LC | A | D | BJ |
| Nectariniidae | *Nectarinia chloropygia* | Sunbird, Olive-bellied | LC | E | S | NG |
|  | *Nectarinia coccinigaster* | Sunbird, Splendid | LC | E | S | BJ,NG |
|  | *Nectarinia cuprea* | Sunbird, Copper | LC | B | S | CD,NG |
|  | *Nectarinia kilimensis* | Sunbird, Bronze | LC | E | S | CD |
|  | *Nectarinia senegalensis* | Sunbird, Scarlet-chested | LC | A | S | BJ,NG |
|  | *Nectarinia superba* | Sunbird, Superb | LC | E | D | BJ |
|  | *Nectarinia verticalis* | Sunbird, Green-headed | LC | E | S | CD,NG |
| Oriolidae | *Oriolus auratus* | Oriole, African Golden | LC | B | S | BJ,NG |
|  | *Oriolus brachyrhyncus* | Oriole, Western Black-headed | LC | F | D | BJ |
|  | *Oriolus larvatus* | Oriole, African Black-headed | LC | F | I | CD |
| Passeridae | *Passer domesticus* | Sparrow, House | LC | A | D | ZA |
|  | *Passer griseus* | Sparrow, Northern Grey-headed | LC | A | S | CD,NG |
|  | *Sporopipes frontalis* | Weaver, Speckle-fronted | LC | H | S | NG |
| Platysteiridae | *Platysteira castanea* | Wattle-eye, Chestnut | LC | E | S | CD |
|  | *Platysteira cyanea* | Wattle-eye, Brown-throated | LC | E | S | NG |
| Ploceidae | *Amblyospiza albifrons* | Weaver, Grosbeak | LC | E | S | CD |
|  | *Anaplectes rubriceps* | Weaver, Red-headed | LC | E | S | NG |
|  | *Bubalornis albirostris* | Buffalo-weaver, White-billed | LC | E | S | BJ |
|  | *Euplectes franciscanus* | Bishop, Orange | LC | F | S | BJ,NG |
|  | *Euplectes* sp*.* | Bishop, unidentified | - | - | - | CI |
|  | *Malimbus nitens* | Malimbe, Gray's | LC | F | S | BJ |
|  | *Malimbus rubricollis* | Malimbe, Red-headed | LC | F | S | NG |
|  | *Malimbus scutatus* | Malimbe, Red-vented | LC | F | S | BJ |
|  | *Ploceus baglafecht* | Weaver, Baglafecht | LC | E | S | CD |
|  | *Ploceus cucullatus* | Weaver, Village | LC | A | S | BF,BJ,NG |
|  | *Ploceus nigerrimus* | Weaver, Vieillot's Black | LC | F | S | BJ,NG |
|  | *Ploceus nigricollis* | Weaver, Black-necked | LC | E | S | CD |
|  | *Ploceus xanthops* | Weaver, Holub's Golden | LC | F | S | CD |
|  | *-* | Weaver, unidentified | - | - | - | CM,TG |
| Pycnonotidae | *Andropadus importunus* | Greenbul, Sombre | LC | H | S | ZA |
|  | *Andropadus latirostris* | Greenbul, Yellow-whiskered | LC | G | S | CD |
|  | *Andropadus tephrolaemus* | Greenbul, Western Mountain | LC | F | S | CD |
|  | *Bleda syndactylus* | Bristlebill, Common | LC | F | S | CD |
|  | *Chlorocichla flavicollis* | Greenbul, Yellow-throated | LC | F | S | BJ,CD,CM |
|  | *Nicator chloris* | Nicator, Yellow-spotted | LC | F | S | BJ |
|  | *Phyllastrephus flavostriatus* | Greenbul, Yellow-streaked | LC | F | S | CD |
|  | *Pycnonotus barbatus* | Bulbul, Common | LC | A | I | BJ,CD,NG,ZA |
| Sturnidae | *Acridotheres tristis* | Myna, Common^e^ | LC | A | I | ZA |
|  | *Cinnyricinclus leucogaster* | Starling, Violet-backed | LC | A | D | NG |
|  | *Lamprotornis chalybaeus* | Glossy-starling, Greater Blue-eared | LC | B | S | NG |
|  | *Lamprotornis nitens* | Glossy-starling, Red-shouldered | LC | E | S | ZA |
|  | *Lamprotornis pulcher* | Starling, Chestnut-bellied | LC | H | S | NG |
|  | *Lamprotornis purpureus* | Glossy-starling, Purple | LC | F | S | BF,BJ |
|  | *Lamprotornis* sp*.* | Glossy-starling, unidentified | - | - | - | BJ,ZA |
|  | *Lamprotornis splendidus* | Starling, Splendid Glossy | LC | F | ? | BJ,NG |
|  | *Onychognathus fulgidus* | Starling, Chestnut-winged | LC | F | D | BJ |
|  | *Onychognathus morio* | Starling, Red-winged | LC | F | I | BJ |
| Sylviidae | *Melocichla mentalis* | Grass-warbler, Moustached | LC | F | S | NG |
|  | *Sylvietta leucophrys* | Crombec, White-browed | LC | F | S | CD |
| Timaliidae | *Turdoides plebejus* | Babbler, Brown | LC | G | S | NG |
| Turdidae | *Neocossyphus poensis* | Ant-thrush, White-tailed | LC | F | ? | BJ,NG |
|  | *Turdus olivaceus* | Thrush, Olive | LC | F | ? | ZA |
|  | *Turdus pelios* | Thrush, African | LC | B | ? | BJ,NG |
| Viduidae | *Vidua macroura* | Whydah, Pin-tailed | LC | A | S | BJ,NG |
|  | *Vidua paradisaea* | Paradise-Whydah, Eastern | LC | D | S | NG |
|  | *Vidua* sp*.* | Paradise-Whydah, unidentified | - | - | - | BJ |
| Zosteropidae | *Zosterops pallidus* | White-eye, Pale | LC | E | ? | ZA |
|  | *Zosterops senegalensis* | White-eye, African Yellow | LC | A | S | CD |
| **PELICANIFORMES** | |  |  |  |  |  |
| Pelecanidae | *Pelecanus onocrotalus* | Pelican, Great White | LC | B | ? | BF,ZA |
|  | *Pelecanus rufescens* | Pelican, Pink-backed | LC | B | S | BJ |
|  | *Pelecanus* sp*.* | Pelican, unidentified | - | - | - | ZA |
| Scopidae | *Scopus umbretta* | Hamerkop | LC | B | S | BF,KE,NG,SZ,ZA |
| Sulidae | *Morus capensis* | Gannet, Cape | VU | F | D | ZA |
| Threskiornithidae | *Bostrychia hagedash* | Ibis, Hadada | LC | A | I | CI,NG,ZA |
|  | *Bostrychia rara* | Ibis, Spot-breasted | LC | H | S | NG |
|  | *Geronticus calvus* | Ibis, Southern Bald | VU | F | D | MA,ZA |
|  | *Threskiornis aethiopicus* | Ibis, African Sacred | LC | A | D | ZA |
| **PICIFORMES** | |  |  |  |  |  |
| Indicatoridae | *Indicator* sp*.* | Honeyguide, unidentified | - | - | - | ZM |
| Picidae | *Campethera abingoni* | Woodpecker, Golden-tailed | LC | E | S | ZA |
|  | *Campethera cailliauti* | Woodpecker, Green-backed | LC | C | S | BJ |
|  | *Campethera punctuligera* | Woodpecker, Fine-spotted | LC | F | S | BJ,NG |
|  | *Dendropicos fuscescens* | Woodpecker, Cardinal | LC | A | S | CD |
|  | *Jynx torquilla*^PAL^ | Wryneck, Eurasian | LC | C | D | NG |
|  | *Mesopicos goertae* | Woodpecker, Grey | LC | A | S | BJ,NG |
|  | *-* | Woodpecker, unidentified | - | - | - | TG,ZA |
| Ramphastidae | *Gymnobucco bonapartei* | Barbet, Grey-throated | LC | F | D | CD |
|  | *Lybius bidentatus* | Barbet, Doubled-toothed | LC | E | S | BF,BJ,NG |
|  | *Lybius dubius* | Barbet, Bearded | LC | G | ? | BF,BJ,NG |
|  | *Lybius torquatus* | Barbet, Black-collared | LC | E | D | CD |
|  | *Lybius vieilloti* | Barbet, Vieillot's | LC | H | ? | NG |
|  | *Pogoniulus bilineatus* | Tinkerbird, Yellow-rumped | LC | B | S | CD |
|  | *Pogoniulus chrysoconus* | Tinkerbird, Yellow-fronted | LC | A | S | BJ,NG |
|  | *Trachyphonus vaillantii* | Barbet, Crested | LC | E | D | ZA |
| **Podicipediformes** | |  |  |  |  |  |
| Podicipedidae | *Tachybaptus ruficollis* | Grebe, Little | LC | B | ? | NG |
| **Procellariiformes** | |  |  |  |  |  |
| Diomedeidae | *Thalassarche cauta* | Albatross, Shy | NT | D | D | ZA |
| **PSITTACIFORMES** | |  |  |  |  |  |
| Psittacidae | *Agapornis pullarius* | Lovebird, Red-headed | LC | E | D | BF,BJ |
|  | *Agapornis taranta* | Lovebird, Black-winged | LC | F | I | ET |
|  | *Poicephalus gulielmi* | Parrot, Red-fronted | LC | F | D | NG |
|  | *Poicephalus senegalus* | Parrot, Senegal | LC | F | S | BF,BJ,NG |
|  | *Poicephalus sp.* | Parrot, unidentified | - | - | - | CM,NG,TG |
|  | *Psittacula krameri* | Parakeet, Rose-ringed^e^ | LC | E | I | BF,BJ,NG |
|  | *Psittacula* sp*.* | Parakeet, unidentified | - | - | - | BF |
|  | *Psittacus erithacus* | Parrot, Grey | VU | F | D | BJ,CD,CM,GH,NG,TG |
| **Pteroclidiformes** | |  |  |  |  |  |
| Pteroclididae | *Pterocles quadricinctus* | Sandgrouse, Four-banded | LC | F | S | NG |
| **Sphenisciformes** | |  |  |  |  |  |
| Spheniscidae | *Spheniscus demersus* | Penguin, African | EN | F | D | ZA |
| **STRIGIFORMES** | |  |  |  |  |  |
| Strigidae | *Asio capensis* | Owl, Marsh | LC | B | S | BJ,ZA |
|  | *Athene noctua* | Owl, Little | LC | A | S | MA |
|  | *Bubo africanus* | Eagle-owl, Spotted | LC | A | S | BJ,CD,LS,NG,ZA |
|  | *Bubo capensis* | Eagle-owl, Cape | LC | G | S | ZA |
|  | *Bubo lacteus* | Eagle-owl, Giant | LC | C | S | NG |
|  | *Bubo poensis* | Eagle-owl, Fraser's | LC | H | S | NG |
|  | *Bubo* sp*.* | Eagle-owl, unidentified | - | - | - | CI,ZA |
|  | *Glaucidium perlatum* | Owlet, Pearl-spotted | LC | B | S | BJ,NG |
|  | *Glaucidium tephronotum* | Owlet, Red-chested | LC | H | S | BJ |
|  | *Otus leucotis* | Scops-owl, White-faced | LC | C | S | BJ,NG |
|  | *Otus scops* | Scops-owl, Common | LC | D | D | BJ |
|  | *Otus senegalensis* | Scops-owl, African | LC | F | S | NG |
|  | *Strix woodfordii* | Wood-owl, African | LC | B | S | BF,BJ,CD,CM,NG,ZA |
|  | *-* | Owl, unidentified | - | - | - | BF,TG,ZA,ZW |
| Tytonidae | *Tyto alba* | Owl, Barn | LC | A | S | BF,BJ,CI,NG,TG,ZA,ZW |
|  | *Tyto capensis* | Grass-owl, African | LC | H | D | ZA |
| **Struthioniformes** | |  |  |  |  |  |
| Struthionidae | *Struthio camelus* | Ostrich | LC | A | D | ER,ET,KE,MZ,NA,NG,SD,SO,SZ,UG,ZA,ZW |
| **Trogoniformes** | |  |  |  |  |  |
| Trogonidae | *Apaloderma narina* | Trogon, Narina | LC | D | S | CD,NG |

^a^ IUCN conservation status: LC=Least Concern, NT=Near Threatened, VU=Vulnerable, EN=Endangered, CR=Critically Endangered)

^b^ Rarity classification: see Table 1

^c^ Population trend: D=declining, S=stable, I=increasing, ?=uncertain

^d^ Country abbreviations: BF=Burkina Faso; BJ=Benin; BW=Botswana; CD=Congo, the DRC; CI=Côte D’Ivoire; CM=Cameroon; ER=Eritrea; ET=Ethiopia; GH=Ghana; KE=Kenya; LS=Lesotho; MA=Morocco; ML=Mali; MW=Malawi; MZ=Mozambique; NA=Namibia; NG=Nigeria; SD=Sudan; SO=Somalia; SZ=Swaziland; TG=Togo; UG=Uganda; ZA=South Africa; ZM=Zambia; ZW=Zimbabwe).

^e^ Exotic species

PAL = Non-breeding Palearctic migrant to Africa.
