# Supplementary material for: Risks to Birds Traded for African Traditional Medicine: A Quantitative Assessment
Source: PLoS One. 2014 Aug 27;9(8):e105397. doi: 10.1371/journal.pone.0105397 (PMC4146541; doi:10.1371/journal.pone.0105397)
Supplement: Tables S2 — a,b,c. Conservation priority groupings, and hence vulnerability to exploitation, of species sold in traditional medicine markets across Africa. Species were sorted into two groups of higher and lower priority and vulnerability according to the K-means cluster analysis. Species are listed within the groups in descending order of their maximum scores for the three standardised variables. (DOC) [file pone.0105397.s003.doc]

**Supporting Information Tables S2. Conservation priority groupings, and hence vulnerability to exploitation, of avian species sold in traditional medicine markets across Africa**. Species are sorted into two groups of higher and lower priority and vulnerability according to the K-means cluster analysis. Species are listed within the groups in descending order of their maximum numerical importance value scores for the three standardised variables.

**Table S2a: Summary of Risk Groups 1 and 2.** Mean variable values per cluster group for 280 species and three variables. ‘Population trend’ was not included as a variable in the analysis because data were not available for 26 species. (* variables used in the cluster analysis)

| Cluster group | Priority/  Vulnerability | Number of species  (*S* = 280) | Mean rarity rank*^a^ | Mean body mass* | Mean no. countries sold in* | Species population trends |
| --- | --- | --- | --- | --- | --- | --- |
| 1 | Higher | 116 | 1.8 ± 0.4  Rarer | 875g ± 1,1661g  Heavier | 1.5 ± 0.8  Fewer markets | Tend to be decreasing |
| 2 | Lower | 164 | 3.4 ± 0.5  More common | 614g ± 1,345g  Lighter | 2.1 ± 0.7  More markets | Tend to be stable |

^a^ Ranks closer to 1 indicate greater inherent rarity, and ranks closer to 4 indicate that species are more common

**Table S2b. Risk Group 1 – Avian species of higher conservation priority (*S*=116).** Taxa tend to be naturally rarer and heavier, and are found in fewer markets (although traders have indicated that they would stock them more if they could obtain them). The populations also tend to be decreasing. This group contains most of the Bucerotiformes (hornbills; 90%), Galliformes (gamebirds; 67%), Gruiformes (cranes and relatives; 67%) and Psittaciformes (parrots; 60%).

| **Order** | **Species** | **Common name** | **Total score**  **(max 3)** |
| --- | --- | --- | --- |
| Falconiformes | *Gyps coprotheres* | Vulture, Cape | 2.13 |
| Gruiformes | *Ardeotis arabs* | Bustard, Arabian | 1.95 |
| Bucerotiformes | *Bucorvus abyssinicus* | Ground-hornbill, Abyssinian | 1.86 |
| Falconiformes | *Torgos tracheliotos* | Vulture, Lappet-faced | 1.82 |
| Gruiformes | *Balearica pavonina* | Crowned-crane, Black | 1.81 |
| Falconiformes | *Stephanoaetus coronatus* | Hawk-eagle, Crowned | 1.67 |
| Falconiformes | *Trigonoceps occipitalis* | Vulture, White-headed | 1.54 |
| Falconiformes | *Gypaetus barbatus* | Lammergeier | 1.52 |
| Ciconiiformes | *Ardea goliath* | Heron, Goliath | 1.52 |
| Bucerotiformes | *Tockus fasciatus* | Hornbill, African Pied | 1.49 |
| Bucerotiformes | *Bucorvus leadbeateri* | Ground-hornbill, Southern | 1.44 |
| Charadriiformes | *Vanellus lugubris* | Lapwing, Senegal | 1.44 |
| Cuculiformes | *Ceuthmochares aereus* | Yellowbill | 1.44 |
| Gruiformes | *Neotis denhami* | Bustard, Denham's | 1.41 |
| Bucerotiformes | *Bycanistes cylindricus* | Hornbill, Brown-cheeked | 1.41 |
| Bucerotiformes | *Crinifer piscator* | Plantain-eater, Western Grey | 1.36 |
| Bucerotiformes | *Ceratogymna elata* | Hornbill, Yellow-casqued | 1.35 |
| Procellariiformes | *Thalassarche cauta* | Albatross, Shy | 1.33 |
| Falconiformes | *Sagittarius serpentarius* | Secretarybird | 1.33 |
| Galliformes | *Guttera pucherani* | Guineafowl, Crested | 1.30 |
| Coraciiformes | *Coracias cyanogaster* | Roller, Blue-bellied | 1.30 |
| Passeriformes | *Cossypha albicapilla* | Robin-chat, White-crowned | 1.29 |
| Sphenisciformes | *Spheniscus demersus* | Penguin, African | 1.23 |
| Psittaciformes | *Psittacus erithacus* | Parrot, Grey | 1.23 |
| Strigiformes | *Bubo poensis* | Eagle-owl, Fraser's | 1.21 |
| Psittaciformes | *Poicephalus senegalus* | Parrot, Senegal | 1.19 |
| Passeriformes | *Lamprotornis splendidus* | Starling, Splendid Glossy | 1.19 |
| Piciformes | *Lybius dubius* | Barbet, Bearded | 1.19 |
| Strigiformes | *Tyto capensis* | Grass-owl, African | 1.19 |
| Ciconiiformes | *Egretta gularis* | Reef-egret, Western | 1.19 |
| Bucerotiformes | *Tauraco corythaix* | Turaco, Knysna | 1.18 |
| Pelecaniformes | *Morus capensis* | Gannet, Cape | 1.18 |
| Gruiformes | *Canirallus oculeus* | Rail, Grey-throated | 1.17 |
| Falconiformes | *Falco alopex* | Kestrel, Fox | 1.17 |
| Galliformes | *Francolinus albogularis* | Francolin, White-throated | 1.17 |
| Bucerotiformes | *Ceratogymna atrata* | Hornbill, Black-casqued | 1.17 |
| Bucerotiformes | *Bycanistes subcylindricus* | Hornbill, Black-and-white-casqued | 1.17 |
| Falconiformes | *Accipiter erythropus* | Sparrowhawk, Red-thighed | 1.16 |
| Strigiformes | *Glaucidium tephronotum* | Owlet, Red-chested | 1.15 |
| Charadriiformes | *Pluvianus aegyptius* | Plover, Egyptian | 1.15 |
| Passeriformes | *Lamprotornis pulcher* | Starling, Chestnut-bellied | 1.15 |
| Piciformes | *Lybius vieilloti* | Barbet, Vieillot's | 1.15 |
| Passeriformes | *Andropadus importunus* | Greenbul, Sombre | 1.15 |
| Upupiformes | *Phoeniculus casteneiceps* | Woodhoopoe, Forest | 1.15 |
| Passeriformes | *Sporopipes frontalis* | Weaver, Speckle-fronted | 1.14 |
| Bucerotiformes | *Corythaeola cristata* | Turaco, Great Blue | 1.14 |
| Gruiformes | *Eupodotis melanogaster* | Bustard, Black-bellied | 1.14 |
| Galliformes | *Francolinus ahantensis* | Francolin, Ahanta | 1.09 |
| Bucerotiformes | *Bycanistes fistulator* | Hornbill, Piping | 1.09 |
| Galliformes | *Francolinus bicalcaratus* | Fancolin, Double-spurred | 1.08 |
| Bucerotiformes | *Musophaga violacea* | Turaco, Violet | 1.07 |
| Gruiformes | *Gallinula chloropus* | Moorhen, Common | 1.07 |
| Cuculiformes | *Centropus leucogaster* | Coucal, Black-throated | 1.07 |
| Bucerotiformes | *Tauraco persa* | Turaco, Guinea | 1.07 |
| Cuculiformes | *Centropus grillii* | Coucal, Black | 1.05 |
| Passeriformes | *Lamprotornis purpureus* | Glossy-starling, Purple | 1.05 |
| Charadriiformes | *Vanellus tectus* | Lapwing, Black-headed | 1.05 |
| Piciformes | *Campethera punctuligera* | Woodpecker, Fine-spotted | 1.04 |
| Passeriformes | *Corvinella corvina* | Shrike, Yellow-billed | 1.04 |
| Coraciiformes | *Merops nubicus* | Bee-eater, Northern Carmine | 1.04 |
| Passeriformes | *Neocossyphus poensis* | Ant-thrush, White-tailed | 1.04 |
| Passeriformes | *Ploceus nigerrimus* | Weaver, Vieillot's Black | 1.04 |
| Coraciiformes | *Merops albicollis* | Bee-eater, White-throated | 1.04 |
| Passeriformes | *Euplectes franciscanus* | Bishop, Orange | 1.04 |
| Strigiformes | *Bubo capensis* | Eagle-owl, Cape | 1.02 |
| Pelecaniformes | *Geronticus calvus* | Ibis, Southern Bald | 1.01 |
| Anseriformes | *Pteronetta hartlaubii* | Duck, Hartlaub's | 0.98 |
| Gruiformes | *Eupodotis senegalensis* | Bustard, White-bellied | 0.98 |
| Anseriformes | *Thalassornis leuconotus* | Duck, White-backed | 0.97 |
| Bucerotiformes | *Bycanistes bucinator* | Hornbill, Trumpeter | 0.96 |
| Gruiformes | *Podica senegalensis* | Finfoot, African | 0.96 |
| Gruiformes | *Himantornis haematopus* | Rail, Nkulengu | 0.96 |
| Ciconiiformes | *Gorsachius leuconotus* | Night-heron, White-backed | 0.94 |
| Columbiiformes | *Columba uncinata* | Pigeon, Afep | 0.94 |
| Bucerotiformes | *Tropicranus albocristatus* | Hornbill, White-crested | 0.92 |
| Falconiformes | *Aviceda cuculoides* | African Baza | 0.92 |
| Psittaciformes | *Poicephalus gulielmi* | Parrot, Red-fronted | 0.92 |
| Bucerotiformes | *Tauraco porphyreolophus* | Turaco, Purple-crested | 0.92 |
| Bucerotiformes | *Tauraco macrorhynchus* | Turaco, Yellow-billed | 0.92 |
| Falconiformes | *Falco chicquera* | Falcon, Red-necked | 0.92 |
| Galliformes | *Ptilopachus petrosus* | Partridge, Stone | 0.91 |
| Falconiformes | *Falco cuvierii* | Hobby, African | 0.91 |
| Pteroclidiformes | *Pterocles quadricinctus* | Sandgrouse, Four-banded | 0.91 |
| Charadriiformes | *Vanellus crassirostris* | Lapwing, Long-toed | 0.91 |
| Charadriiformes | *Vanellus armatus* | Lapwing, Blacksmith | 0.91 |
| Ciconiiformes | *Ixobrychus sturmii* | Bittern, Dwarf | 0.91 |
| Passeriformes | *Onychognathus morio* | Starling, Red-winged | 0.91 |
| Passeriformes | *Onychognathus fulgidus* | Starling, Chestnut-winged | 0.90 |
| Falconiformes | *Chelictinia riocourii* | Kite, African Swallow-tailed | 0.90 |
| Bucerotiformes | *Tockus camurus* | Hornbill, Red-billed Dwarf | 0.90 |
| Bucerotiformes | *Tockus hartlaubi* | Hornbill, Black Dwarf | 0.90 |
| Strigiformes | *Otus scops* | Scops-owl, Common | 0.90 |
| Passeriformes | *Turdus olivaceus* | Thrush, Olive | 0.90 |
| Trogoniformes | *Apaloderma narina* | Trogon, Narina | 0.90 |
| Strigiformes | *Otus senegalensis* | Scops-owl, African | 0.90 |
| Passeriformes | *Coracina pectoralis* | Cuckooshrike, White-breasted | 0.90 |
| Passeriformes | *Chlorocichla flavicollis* | Greenbul, Yellow-throated | 0.90 |
| Passeriformes | *Oriolus brachyrhyncus* | Oriole, Western Black-headed | 0.90 |
| Caprimulgiformes | *Caprimulgus nigriscapularis* | Nightjar, Black-shouldered | 0.90 |
| Passeriformes | *Nicator chloris* | Nicator, Yellow-spotted | 0.90 |
| Gruiformes | *Sarothrura pulchra* | Flufftail, White-spotted | 0.90 |
| Coraciiformes | *Merops malimbicus* | Bee-eater, Rosy | 0.90 |
| Galliformes | *Coturnix chinensis* | Quail, Blue | 0.90 |
| Passeriformes | *Malimbus rubricollis* | Malimbe, Red-headed | 0.90 |
| Passeriformes | *Malimbus nitens* | Malimbe, Gray's | 0.90 |
| Passeriformes | *Cossypha niveicapilla* | Robin-chat, Snowy-crowned | 0.90 |
| Passeriformes | *Melocichla mentalis* | Grass-warbler, Moustached | 0.90 |
| Passeriformes | *Dryoscopus gambensis* | Puffback, Northern | 0.90 |
| Passeriformes | *Cossypha natalensis* | Robin-chat, Red-capped | 0.90 |
| Passeriformes | *Malimbus scutatus* | Malimbe, Red-vented | 0.90 |
| Passeriformes | *Hypergerus atriceps* | Warbler, Oriole | 0.90 |
| Passeriformes | *Spermophaga haematina* | Bluebill, Western | 0.90 |
| Passeriformes | *Vidua paradisaea* | Paradise-Whydah, Eastern | 0.90 |
| Passeriformes | *Terpsiphone rufiventer* | Paradise-Flycatcher, Black-headed | 0.89 |
| Passeriformes | *Estrilda trogolodytes* | Waxbill, Black-rumped | 0.89 |

**Table S2c. Risk Group 2 – Avian species of lower conservation priority (*S*=164).** Taxa tend to be naturally more common and lighter (body mass), and are found in more markets. The populations also tend to be stable or increasing. This group contains most of the Columbiformes (doves; 89%), Ciconiiformes (storks; 79%), Coraciiformes (kingfishers; 76%), Cuculiformes (cuckoos and relatives; 73%), Falconiformes (diurnal birds of prey; 69%), and Passeriformes (perching birds; 59%).

| **Order** | **Species** | **Common name** | **Total score**  **(max 3)** |
| --- | --- | --- | --- |
| Falconiformes | *Gyps rueppellii* | Vulture, Rueppell's | 1.80 |
| Pelecaniformes | *Pelecanus onocrotalus* | Pelican, Great White | 1.79 |
| Falconiformes | *Gyps africanus* | Vulture, White-backed | 1.58 |
| Falconiformes | *Haliaeetus vocifer* | Fish-eagle, African | 1.37 |
| Strigiformes | *Tyto alba* | Owl, Barn | 1.29 |
| Pelecaniformes | *Pelecanus rufescens* | Pelican, Pink-backed | 1.23 |
| Ciconiiformes | *Leptoptilos crumeniferus* | Stork, Marabou | 1.22 |
| Falconiformes | *Necrosyrtes monachus* | Vulture, Hooded | 1.21 |
| Ciconiiformes | *Nycticorax nycticorax* | Night-heron, Black-crowned | 1.16 |
| Anseriformes | *Plectropterus gambensis* | Goose, Spur-winged | 1.12 |
| Galliformes | *Numida meleagris* | Guineafowl, Helmeted | 1.10 |
| Strigiformes | *Strix woodfordii* | Wood-owl, African | 1.10 |
| Bucerotiformes | *Tockus erythrorhynchus* | Hornbill, Red-billed | 1.09 |
| Gruiformes | *Porphyrio porphyrio* | Swamphen, Purple | 1.03 |
| Falconiformes | *Neophron percnopterus* | Vulture, Egyptian | 1.01 |
| Anseriformes | *Dendrocygna viduata* | Whistling-duck, White-faced | 1.01 |
| Ciconiiformes | *Scopus umbretta* | Hamerkop | 0.97 |
| Cuculiformes | *Centropus senegalensis* | Coucal, Senegal | 0.95 |
| Falconiformes | *Lophaetus occipitalis* | Eagle, Long-crested | 0.94 |
| Psittaciformes | *Psittacula krameri* | Parakeet, Rose-ringed* | 0.94 |
| Gruiformes | *Amaurornis flavirostra* | Crake, Black | 0.94 |
| Piciformes | *Lybius bidentatus* | Barbet, Doubled-toothed | 0.94 |
| Coraciiformes | *Alcedo cristata* | Kingfisher, Malachite | 0.93 |
| Passeriformes | *Turdoides plebejus* | Babbler, Brown | 0.90 |
| Ciconiiformes | *Mycteria ibis* | Stork, Yellow-billed | 0.88 |
| Strigiformes | *Bubo lacteus* | Eagle-owl, Giant | 0.88 |
| Ciconiiformes | *Bubulcus ibis* | Egret, Cattle | 0.86 |
| Falconiformes | *Terathopius ecaudatus* | Bateleur | 0.85 |
| Falconiformes | *Circaetus cinereus* | Snake-eagle, Brown | 0.84 |
| Ciconiiformes | *Ardea cinerea* | Heron, Grey | 0.83 |
| Coraciiformes | *Coracias abyssinicus* | Roller, Abyssinian | 0.83 |
| Coraciiformes | *Megaceryle maxima* | Kingfisher, Giant | 0.82 |
| Charadriiformes | *Burhinus senegalensis* | Thick-knee, Senegal | 0.82 |
| Strigiformes | *Asio capensis* | Owl, Marsh | 0.82 |
| Pelecaniformes | *Bostrychia hagedash* | Ibis, Hadada | 0.81 |
| Falconiformes | *Falco ardosiaceus* | Kestrel, Grey | 0.81 |
| Strigiformes | *Otus leucotis* | Scops-owl, White-faced | 0.81 |
| Coraciiformes | *Coracias naevia* | Roller, Rufus-crowned | 0.80 |
| Cuculiformes | *Centropus superciliosus* | Coucal, White-browed | 0.80 |
| Falconiformes | *Aquila rapax* | Eagle, Tawny | 0.80 |
| Gruiformes | *Gallinula angulata* | Moorhen, Lesser | 0.80 |
| Falconiformes | *Gypohierax angolensis* | Vulture, Palm-nut | 0.80 |
| Cuculiformes | *Clamator levaillantii* | Cuckoo, Levaillant's | 0.80 |
| Galliformes | *Coturnix coturnix* | Quail, Common | 0.80 |
| Coraciiformes | *Ceryle rudis* | Kingfisher, Pied | 0.79 |
| Strigiformes | *Glaucidium perlatum* | Owlet, Pearl-spotted | 0.79 |
| Passeriformes | *Malaconotus blanchoti* | Bush-shrike, Grey-headed | 0.79 |
| Ciconiiformes | *Ciconia abdimii* | Stork, Abdim's | 0.79 |
| Galliformes | *Coturnix delegorguei* | Quail, Harlequin | 0.79 |
| Upupiformes | *Phoeniculus purpureus* | Woodhoopoe, Green | 0.79 |
| Passeriformes | *Oriolus auratus* | Oriole, African Golden | 0.79 |
| Passeriformes | *Turdus pelios* | Thrush, African | 0.79 |
| Caprimulgiformes | *Macrodipteryx longipennis* | Nightjar, Standard-winged | 0.79 |
| Caprimulgiformes | *Caprimulgus climacurus* | Nightjar, Long-tailed | 0.79 |
| Psittaciformes | *Agapornis pullarius* | Lovebird, Red-headed | 0.79 |
| Passeriformes | *Nectarinia coccinigaster* | Sunbird, Splendid | 0.79 |
| Passeriformes | *Hirundo aethiopica* | Swallow, Ethiopian | 0.79 |
| Falconiformes | *Buteo rufofuscus* | Buzzard, Jackal | 0.78 |
| Ciconiiformes | *Anastomus lamelligerus* | Openbill, African | 0.77 |
| Falconiformes | *Circaetus cinerascens* | Snake-eagle, Banded | 0.76 |
| Ciconiiformes | *Ardea purpurea* | Heron, Purple | 0.76 |
| Strigiformes | *Bubo africanus* | Eagle-owl, Spotted | 0.75 |
| Falconiformes | *Polyboroides typus* | Harrier-hawk, African | 0.75 |
| Anseriformes | *Anas undulata* | Duck, Yellow-billed | 0.74 |
| Passeriformes | *Corvus albicollis* | Raven, White-necked | 0.74 |
| Falconiformes | *Melierax metabates* | Chanting-Goshawk, Dark | 0.73 |
| Anseriformes | *Dendrocygna bicolor* | Whistling-duck, Fulvous | 0.72 |
| Falconiformes | *Circus aeruginosus* | Marsh-harrier, Western | 0.72 |
| Falconiformes | *Elanus caeruleus* | Kite, Black-winged | 0.71 |
| Charadriiformes | *Sterna caspia* | Tern, Caspian | 0.71 |
| Passeriformes | *Corvus capensis* | Crow, Cape | 0.70 |
| Columbiiformes | *Streptopelia semitorquata* | Dove, Red-eyed | 0.70 |
| Bucerotiformes | *Tockus nasutus* | Hornbill, African Grey | 0.70 |
| Upupiformes | *Upupa epops* | Hoopoe, Eurasian | 0.69 |
| Coraciiformes | *Halcyon senegalensis* | Kingfisher, Woodland | 0.68 |
| Passeriformes | *Tchagra senegalus* | Tchagra, Black-crowned | 0.68 |
| Passeriformes | *Pycnonotus barbatus* | Bulbul, Common | 0.68 |
| Passeriformes | *Ploceus cucullatus* | Weaver, Village | 0.68 |
| Passeriformes | *Prionops plumatus* | Helmet-Shrike, White | 0.68 |
| Charadriiformes | *Larus cirrocephalus* | Gull, Grey-headed | 0.68 |
| Charadriiformes | *Recurvirostra avosetta* | Avocet, Pied | 0.68 |
| Ciconiiformes | *Ardeola ralloides* | Heron, Squacco | 0.67 |
| Columbiiformes | *Treron waalia* | Green-pigeon, Bruce's | 0.67 |
| Falconiformes | *Accipiter tachiro* | Goshawk, African | 0.67 |
| Charadriiformes | *Vanellus senegallus* | Lapwing, Wattled | 0.67 |
| Charadriiformes | *Actophilornis africanus* | Jacana, African | 0.66 |
| Charadriiformes | *Vanellus albiceps* | Lapwing, White-headed | 0.66 |
| Podicipediformes | *Tachybaptus ruficollis* | Grebe, Little | 0.66 |
| Falconiformes | *Melierax gabar* | Goshawk, Gabar | 0.66 |
| Charadriiformes | *Vanellus coronatus* | Lapwing, Crowned | 0.66 |
| Gruiformes | *Porphyrio alleni* | Gallinule, Allen's | 0.66 |
| Passeriformes | *Ptilostomus afer* | Piapiac | 0.66 |
| Gruiformes | *Crecopsis egregia* | Crake, African | 0.66 |
| Passeriformes | *Lamprotornis nitens* | Glossy-starling, Red-shouldered | 0.65 |
| Passeriformes | *Lamprotornis chalybaeus* | Glossy-starling, Greater Blue-eared | 0.65 |
| Charadriiformes | *Glareola pratincola* | Pratincole, Collared | 0.65 |
| Coraciiformes | *Halcyon malimbica* | Kingfisher, Blue-breasted | 0.65 |
| Piciformes | *Trachyphonus vaillantii* | Barbet, Crested | 0.65 |
| Piciformes | *Campethera abingoni* | Woodpecker, Golden-tailed | 0.65 |
| Coraciiformes | *Halcyon albiventris* | Kingfisher, Brown-hooded | 0.65 |
| Passeriformes | *Laniarius ferrugineus* | Boubou, Southern | 0.65 |
| Passeriformes | *Bubalornis albirostris* | Buffalo-weaver, White-billed | 0.65 |
| Passeriformes | *Laniarius aethiopicus* | Boubou, Ethiopian | 0.65 |
| Passeriformes | *Laniarius barbarus* | Gonolek, Common | 0.65 |
| Piciformes | *Campethera cailliauti* | Woodpecker, Green-backed | 0.65 |
| Passeriformes | *Melaenornis edolioides* | Flycatcher, Northern Black | 0.65 |
| Passeriformes | *Motacilla aguimp* | Wagtail, African Pied | 0.65 |
| Passeriformes | *Melaenornis pammelaina* | Flycatcher, Southern Black | 0.65 |
| Passeriformes | *Anaplectes rubriceps* | Weaver, Red-headed | 0.65 |
| Apodiformes | *Apus caffer* | Swift, White-rumped | 0.65 |
| Upupiformes | *Rhinopomastus aterrimus* | Scimitarbill, Black | 0.65 |
| Passeriformes | *Nectarinia superba* | Sunbird, Superb | 0.64 |
| Passeriformes | *Platysteira cyanea* | Wattle-eye, Brown-throated | 0.64 |
| Passeriformes | *Nectarinia verticalis* | Sunbird, Green-headed | 0.64 |
| Passeriformes | *Terpsiphone viridis* | Paradise-Flycatcher, African | 0.64 |
| Passeriformes | *Hirundo smithii* | Swallow, Wire-tailed | 0.64 |
| Passeriformes | *Zosterops pallidus* | White-eye, Pale | 0.64 |
| Passeriformes | *Nectarinia cuprea* | Sunbird, Copper | 0.64 |
| Passeriformes | *Nectarinia chloropygia* | Sunbird, Olive-bellied | 0.64 |
| Falconiformes | *Milvus migrans* | Kite, Black | 0.62 |
| Falconiformes | *Buteo auguralis* | Buzzard, Red-necked | 0.61 |
| Charadriiformes | *Burhinus capensis* | Thick-knee, Spotted | 0.59 |
| Columbiiformes | *Columba guinea* | Pigeon, Speckled | 0.57 |
| Falconiformes | *Kaupifalco monogrammicus* | Buzzard, Lizard | 0.57 |
| Ciconiiformes | *Butorides striata* | Heron, Striated | 0.56 |
| Falconiformes | *Falco tinnunculus* | Kestrel, Common | 0.56 |
| Pelecaniformes | *Threskiornis aethiopicus* | Ibis, African Sacred | 0.55 |
| Columbiiformes | *Streptopelia capicola* | Dove, Ring-necked | 0.55 |
| Falconiformes | *Accipiter badius* | Shikra | 0.55 |
| Cuculiformes | *Cuculus gularis* | Cuckoo, African | 0.55 |
| Passeriformes | *Dicrurus adsimilis* | Drongo, Fork-tailed | 0.54 |
| Piciformes | *Mesopicos goertae* | Woodpecker, Grey | 0.54 |
| Coraciiformes | *Halcyon leucocephala* | Kingfisher, Grey-headed | 0.54 |
| Passeriformes | *Lanius collaris* | Fiscal, Common | 0.54 |
| Cuculiformes | *Chrysococcyx caprius* | Cuckoo, Didric | 0.54 |
| Passeriformes | *Vidua macroura* | Whydah, Pin-tailed | 0.54 |
| Piciformes | *Pogoniulus chrysoconus* | Tinkerbird, Yellow-fronted | 0.54 |
| Coraciiformes | *Ceyx pictus* | Pygmy-kingfisher, African | 0.54 |
| Passeriformes | *Nectarinia senegalensis* | Sunbird, Scarlet-chested | 0.54 |
| Passeriformes | *Lagonostica senegala* | Firefinch, Red-billed | 0.54 |
| Ciconiiformes | *Ardea melanocephala* | Heron, Black-headed | 0.51 |
| Ciconiiformes | *Casmerodius albus* | Egret, Great | 0.49 |
| Falconiformes | *Aquila wahlbergi* | Eagle, Wahlberg's | 0.49 |
| Falconiformes | *Falco biarmicus* | Falcon, Lanner | 0.46 |
| Ciconiiformes | *Egretta garzetta* | Egret, Little | 0.45 |
| Ciconiiformes | *Mesophoyx intermedia* | Egret, Intermediate | 0.44 |
| Columbiiformes | *Columba livia* | Pigeon, Rock | 0.43 |
| Falconiformes | *Butastur rufipennis* | Buzzard, Grasshopper | 0.43 |
| Columbiiformes | *Treron calvus* | Green-pigeon, African | 0.41 |
| Cuculiformes | *Clamator glandarius* | Cuckoo, Great Spotted | 0.41 |
| Charadriiformes | *Rhinoptilus chalcopterus* | Courser, Bronze-winged | 0.41 |
| Coraciiformes | *Eurystomis glaucurus* | Roller, Broad-billed | 0.40 |
| Columbiiformes | *Stigmatopelia senegalensis* | Dove, Laughing | 0.40 |
| Cuculiformes | *Cuculus clamosus* | Cuckoo, Black | 0.40 |
| Cuculiformes | *Clamator jacobinus* | Cuckoo, Pied | 0.40 |
| Columbiiformes | *Turtur afer* | Wood-dove, Blue-spotted | 0.40 |
| Coliiformes | *Colius striatus* | Mousebird, Speckled | 0.40 |
| Passeriformes | *Cinnyricinclus leucogaster* | Starling, Violet-backed | 0.40 |
| Coraciiformes | *Halcyon chelicuti* | Kingfisher, Striped | 0.40 |
| Passeriformes | *Passer domesticus* | Sparrow, House | 0.40 |
| Passeriformes | *Passer griseus* | Sparrow, Northern Grey-headed | 0.40 |
| Passeriformes | *Hirundo abyssinica* | Striped-swallow, Lesser | 0.39 |
| Apodiformes | *Apus affinis* | Swift, Little | 0.39 |
| Coraciiformes | *Merops pusillus* | Bee-eater, Little | 0.39 |
| Passeriformes | *Prinia subflava* | Prinia, Tawny-flanked | 0.39 |
