# Supplementary material for: Risks to Birds Traded for African Traditional Medicine: A Quantitative Assessment
Source: PLoS One. 2014 Aug 27;9(8):e105397. doi: 10.1371/journal.pone.0105397 (PMC4146541; doi:10.1371/journal.pone.0105397)
Supplement: Table S3 — Mean EOOs for traded bird species in the eight Rabinowitz and four rarity categories (corresponding with the classes in Table 1), where A and 4 are the most common species, and H and 1 are most rare species (excludes PALs and exotics). Birds with an EOO >6,790,00 km2 were assigned to classes A to D. (DOCX) [file pone.0105397.s004.docx]

**Supporting Information Table S3. Mean EOOs for traded bird species in the eight Rabinowitz and four rarity categories** (corresponding with the classes in Table 1). Species in class A and ranked 4 are the most common, and species in class H and ranked 1 are the most rare (excludes PALs and exotics).

The mean EOO of species within each rarity class decreases with increasing species rarity (from A to H). The EOO of species grouped according to the four simplified rarity ranks also decreases with increasing rarity. The Tukey post-hoc test established that mean EOOs for species ranked 1 and 2 were not significantly different from each other (*P*=0.79), but had significantly smaller ranges than species ranked 3 and 4 (*P*<0.001; Tukey HSD F_7, 275_=31.63, *P*<0.001). The mean EOO of group 4 was significantly larger than groups 1, 2 and 3 (*P*<0.001).

|  | No. of species *(N)* | Mean (km^2^) | Std. Dev. | Minimum | Maximum |
| --- | --- | --- | --- | --- | --- |
| Rabinowitz classes | |  |  |  |  |
| A | 67 | 18,392,836 | 12,945,412 | 7,040,000 | 63,300,000 |
| B | 54 | 14,793,889 | 7,621,278 | 6,790,000 | 41,200,000 |
| C | 13 | 11,163,077 | 3,487,603 | 6,840,000 | 18,700,000 |
| D | 20 | 10,936,500 | 4,238,174 | 6,910,000 | 23,900,000 |
| E | 28 | 3,667,179 | 1,581,507 | 721,000 | 6,670,000 |
| F | 66 | 3,059,758 | 1,644,830 | 130,000 | 6,250,000 |
| G | 5 | 3,049,800 | 1,865,487 | 969,000 | 5,090,000 |
| H | 30 | 2,866,733 | 1,821,814 | 125,000 | 6,620,000 |
|  |  |  |  |  |  |
| Rarity ranks |  |  |  |  |  |
| 4 | 67 | 18,392,836 | 12,945,412 | 7,040,000 | 63,300,000 |
| 3 | 95 | 11,017,589 | 7,701,254 | 721,000 | 41,200,000 |
| 2 | 91 | 4,790,363 | 4,081,630 | 130,000 | 23,900,000 |
| 1 | 30 | 2,866,733 | 1,821,814 | 125,000 | 6,620,,000 |
| *Total* | 283^a^ | 9,897,230 | 9,792,590 | 125,000 | 63,300,000 |

^a^ No EOO available for five species
