# Supplementary material for: Risks to Birds Traded for African Traditional Medicine: A Quantitative Assessment
Source: PLoS One. 2014 Aug 27;9(8):e105397. doi: 10.1371/journal.pone.0105397 (PMC4146541; doi:10.1371/journal.pone.0105397)
Supplement: Table S4 — Comparison between the number of species per order recorded as being used for traditional medicine in Africa (this study), Brazil (from [9], [10], [75], [76]) and India (from [11], [78]–[80]). Taxa arranged according to the orders common. (DOCX) [file pone.0105397.s005.docx]

**Supporting Information Table S4.** Comparison between the number of species per order recorded as being used for traditional medicine in Africa (this study), South Africa [18–20,22,27,28,37], Brazil [9,10,75,76] and India [11,78–80].

|  | Africa  *N*=354 species  25 countries | South Africa  *N*=118 species ^a^ | Brazil  *N*=54 species ^b^ | India  *N*=31 species |
| --- | --- | --- | --- | --- |
| Passeriformes (Perching birds) | 107 | 17 | 8 | **5** |
| Falconiformes (Diurnal birds of prey) | 45 | 14 | 5 | 2 |
| Charadriiformes (Gulls & relatives) | 19 | 7 | 1 | 1 |
| Galliformes (Gamebirds) | 13 | 4 | 7 | **5** |
| Cuculiformes (Cuckoos & relatives) | 13 | 2 | 2 | 1 |
| Columbiformes (Doves & pigeons) | 10 | 5 | 7 | 2 |
| Ciconiiformes (Storks) | 20 | 5 | 4 |  |
| Gruiiformes (Cranes & relatives) | 17 | 3 | 2 |  |
| Piciformes (Woodpeckers & relatives) | 15 | 2 | 5 |  |
| Anseriformes (Waterfowl) | 7 | 2 | 2 |  |
| Psittaciformes (Parrots) | 6 |  | 2 |  |
| Caprimulgiformes (Nightjars & relatives) | 5 |  | 1 |  |
| Apodiformes (Swifts & relatives) | 2 |  | 1 |  |
| Struthioniformes (Ratites) | 1 | 1 | 1 |  |
| Coraciiformes (Kingfishers & relatives) | 24 | 5 |  | 3 |
| Strigiformes (Owls) | 14 | 6 |  | 6 |
| Bucerotiformes (Hornbills) | 14 | 3 |  | 5 |
| Pelecaniformes (Pelicans & relatives) | 8 | 6 |  | 1 |
| Musophagiformes (Turacos) | 8 | 2 |  |  |
| Coliiformes (Mousebirds) | 1 | 1 |  |  |
| Podicipediformes (Grebes) | 1 |  |  |  |
| Procellariiformes (Albatrosses & relatives) | 1 | 1 |  |  |
| Pteroclidiformes (Soundgrouses) | 1 |  |  |  |
| Sphenisciformes (Penguins) | 1 | 1 |  |  |
| Trogoniformes (Trogons & relatives) | 1 |  |  |  |
| Tinamiformes (Tinamous) |  |  | 6 |  |

^a^ 118 taxa identified to species in South Africa (including exotics; excluding 15 taxa identified to genus only and 9 taxa identified to family only). Of these, 80 species were recorded in TM markets, including 54 species in the large TM market in Johannesburg [20].

^b^ Bezerra *et al.* [76] report that at least 53 bird species are used in folk medicinal practices in Brazil when combining their study with that of Alves *et al.* [9] and Costa-Neto & Alves [77]. We counted 54 species using the same sources.
